# Statistical Analysis of the Impact of COVID-19 on PM_2.5_ Concentrations in Downtown Quito during the Lockdowns in 2020

**DOI:** 10.3390/s22228985

**Published:** 2022-11-20

**Authors:** Wilmar Hernandez, Francisco José Arqués-Orobón, Vicente González-Posadas, José Luis Jiménez-Martín, Paul D. Rosero-Montalvo

**Affiliations:** 1 Facultad de Ingenieria y Ciencias Aplicadas, Universidad de Las Americas, Quito 170124, Ecuador; 2 Departamento de Teoria de la Señal y Comunicaciones, ETSIS de Telecomunicacion, Universidad Politecnica de Madrid, 28031 Madrid, Spain; 3 Computer Science Department, IT University of Copenhagen, 2300 Copenhagen, Denmark

**Keywords:** correlation coefficients, COVID-19, estimation quality, multidimensional scaling, PM_2.5_, principal component analysis

## Abstract

In this paper, a comparative analysis between the PM${}_{2.5}$ concentration in downtown Quito, Ecuador, during the COVID-19 pandemic in 2020 and the previous five years (from 2015 to 2019) was carried out. Here, in order to fill in the missing data and achieve homogeneity, eight datasets were constructed, and 35 different estimates were used together with six interpolation methods to put in the estimated value of the missing data. Additionally, the quality of the estimations was verified by using the sum of squared residuals and the following correlation coefficients: Pearson’s *r*, Kendall’s τ, and Spearman’s ρ. Next, feature vectors were constructed from the data under study using the wavelet transform, and the differences between feature vectors were studied by using principal component analysis and multidimensional scaling. Finally, a robust method to impute missing data in time series and characterize objects is presented. This method was used to support the hypothesis that there were significant differences between the PM${}_{2.5}$ concentration in downtown Quito in 2020 and 2015–2019.

## 1. Introduction

This paper analyzes the data of the PM${}_{2.5}$ concentration (particulate matter with a particle size less than 2.5 μm) [1,2,3,4] in downtown Quito, Ecuador, during the years 2015–2020. Here, the PM${}_{2.5}$ concentration during 2020 was compared with this type of concentration during 2015–2019. The measurements were taken by using the FH62C14 continuous ambient particulate monitor (https://assets.thermofisher.com/TFS-Assets/null%7Cnull/Package-Inserts/EPM-manual-FH62C14.pdf (accessed on 30 September 2022)) of the air quality monitoring station located in Quito’s historic center at García Moreno Street 751 and Sucre (http://www.quitoambiente.gob.ec/index.php/centro (accessed on 30 September 2022)). This air quality monitoring station is part of the Quito Metropolitan Network of Atmospheric Monitoring (QMNAM) and is an urban background station. As in [5,6], here, the historical PM${}_{2.5}$ concentration data were obtained from a website. In this case, it was the QMNAM website. However, as it could happen that sometimes this website does not work properly due to any reason, then an official of QMNAM also gave us the data for this research (as in [7]).

As is well known, hundreds of millions of people around the world have been infected by COVID-19, and several million have died [8,9,10,11,12,13,14]. Therefore, in order to reduce the transmission of COVID-19, governments have taken measures that have proven to be very effective [15,16]. For example, these measures include social distancing, confinements, banning crowds, banning or reducing both national and international travel, and the lockdown of regions in the most affected countries, among others [17,18]. These measures have reduced human activity, reducing industrial production, causing factories to close, and reducing transport, especially by road, among other things. However, fortunately, the above has also reduced the type of air pollution that is caused by the burning of fossil fuels and the production of energy with other nonrenewable sources. For example, the scientific literature on the subject has reported that the PM${}_{2.5}$ concentration, among other air pollutants, has significantly decreased in cities where the population has been in a lockdown for a long time [16,19,20,21,22,23,24,25,26,27,28,29].

In this sense, the main objective of this paper was to compare the measurements of PM${}_{2.5}$ concentration taken in downtown Quito (i.e., Quito’s historic center) in 2020 with the ones taken from 2015 to 2019. Here, the classification of PM${}_{2.5}$ concentration was performed using pattern recognition [30]. Other applications of pattern recognition to solve air quality issues are as follows. In [31], pattern recognition was used to recognize target gases and estimate their concentration. The technique used in [31] was based on principal component analysis (PCA) and the k-nearest neighbors algorithm. In addition, in order to achieve stable and accurate measurements of air pollution based on image processing, a dual-channel 3D convolution network was proposed in [32]. In this case, pattern recognition is of paramount importance because patterns convey relevant information for precise classification. Additionally, in [33], a modeling framework was presented to create power sector emission scenarios for use in air quality models. Here, weather patterns were taken into account to build emissions scenarios. Furthermore, in [34], a pattern recognition tool based on semi-supervised learning was used in artificial olfaction research, where an adaptive strategy was conceived of and tested on an air pollution dataset. Moreover, a linear discriminant analysis pattern recognition tool was used in [35] to evaluate multidimensional data, in the detection of volatile organic compound (VOC) concentrations. In [35], the VOCs’ concentration was detected by using a SiC field-effect transistor with a platinum gate aimed at gas sensing. The use of the above-mentioned pattern recognition tool showed that it was possible to discriminate some indoor air pollutants (e.g., formaldehyde, naphthalene, and benzene). Besides, to improve prediction accuracy, an end-to-end recurrent neural network framework was presented in [36]. In this case, the neural network was evaluated on multivariate air quality datasets, among others.

The use of pattern recognition tools in the present paper is different from the above-mentioned ones. First, the hypothesis to be tested is that the lockdown during 2020, in downtown Quito, produced a significant drop in PM${}_{2.5}$ concentration throughout that year when compared with the previous five years. In addition, the method to argue for this difference is to consider only the PM${}_{2.5}$ concentration and obtain a feature vector [30] for each year, using the statistics of the elements of the wavelet decomposition [37] of the data matrix by year. Then, using both PCA and multidimensional scaling (MDS) [38,39,40], the differences between the feature vectors were analyzed. In short, the main goal of this analysis was to prove that there were differences between 2020 and 2015–2019 and that these differences were bigger from the second trimester, when the lockdown began, in March 2020.

The type of classification of PM${}_{2.5}$ concentration used in this paper was based on both the identification of the object by means of feature vectors and a measure of separation between feature vectors, which was calculated using some kind of metric.

Furthermore, the characteristics of the data under study were obtained using digital imaging procedures [41]. Therefore, the size of the different sets of observations was adapted to make nonoverlapping partitions of sequences whose lengths were powers of two [42,43]. Moreover, since the sampling period was 1 h (http://www.quitoambiente.gob.ec/images/Secretaria_Ambiente/red_monitoreo/informacion/Informe_Calidad_Aire_2017.pdf (accessed on 30 September 2022)), in each non-leap year, there should be 8760 observations. However, as there were many missing data, it was decided to separate the observations for each year into semesters in order to group them into sequences of size $2^{12}$. This means having 4096 observations per semester.

In this research, the data were characterized by means of the statistics of the marginal and joint distributions. Moreover, the statistics of the joint distributions were based on Markov models in which the model was also characterized by interactions between neighboring data [44,45,46]. Lastly, the comparison between feature vectors was performed using both PCA and MDS [47,48,49].

Another type of statistical analysis of PM${}_{2.5}$ concentration was the one carried out in [5]. There, the authors focused on obtaining a linear model based on statistical methods [50] that describe the relationship between indoor and outdoor PM${}_{2.5}$ in buildings. To do this, as is often done when trying to find a solution to an engineering problem, the data were treated as a time series [50,51,52]. In addition, indoor PM${}_{2.5}$ was treated as a random variable with a Gaussian distribution, and four models were tested to approximate the relationship between indoor and outdoor PM${}_{2.5}$ data in buildings. In summary, the building was treated as a dynamic system whose input–output relationship, according to the authors of [5] is best described as a Box–Jenkins model [50].

One more research work aimed at the analysis of contaminants in buildings is the one presented in [53]. There, the authors highlighted the importance of indoor air quality [54,55,56,57] and the safety of the occupants, the latter being affected by an accident or a terrorist attack. In this sense, chemical and biological agents are a potential issue to consider. In [53], a cognitive monitoring system that consisted of three layers was proposed to detect and isolate both sensor failures and contaminants in buildings. In addition, statistical hypothesis tests were performed to detect contaminants and consequently carry out the statistical analysis of their distributions. The idea was to try to discriminate between contaminants and sensor failures and to isolate areas where contaminants have been found or sensors have failed.

Other research in which the statistical analysis of PM${}_{2.5}$ concentration is of paramount importance is [7]. In [7], multivariate regression was used to create a cellular automata model aimed at analyzing both PM${}_{2.5}$ diffusion and generation. In addition, regarding PM${}_{2.5}$ concentration simulation and prediction, the effectiveness of such a model was shown. Furthermore, visualization and correlation analysis were used to find patterns, and the model was trained by using several functions. Moreover, the model did not have a central control, and depended on rules and statistical learning. On the other hand, the richness of the data limited the prediction capacity of the model, and it did not work satisfactorily for extreme climate events. Finally, hidden Markov models and machine learning methods were used in [51,58] to analyze PM${}_{2.5}$ concentration data in Xiamen, Fujian, China.

However, despite the large number of PM${}_{2.5}$ concentration statistical analysis works reported in the literature, we think that there is still room for creating techniques that allow this type of data to be treated as objects that are similar and different at the same time.

Therefore, the raison d’être of the analysis carried out in this paper was that it was necessary to find important differences between possibly similar objects, but having only the data series to carry out the analysis. Furthermore, trying to contribute to fill the gaps that exist in the areas of missing value imputation and object characterization, a robust method to refine the measurement of differences and their presentation is proposed. The results showed that the measures taken to reduce the transmission of COVID-19 also reduced the PM${}_{2.5}$ concentration in downtown Quito.

The organization of this paper is as follows. Section 2 presents the materials and methods. The results are shown in Section 3 along with the discussion. Finally, the conclusions are given in Section 4.

## 2. Materials and Methods

**Figure 1:**
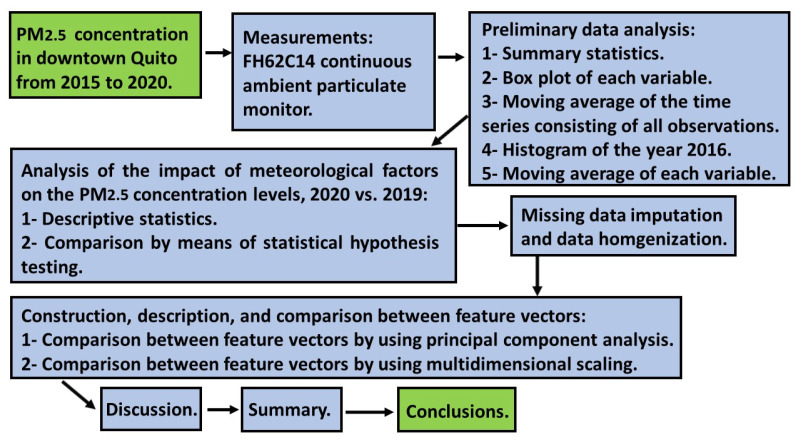
Flowchart of the analysis process that was followed in this research.

The main objective of this section is to explain what procedures, approaches, designs, and treatments were carried out in this research. In addition, in order to graphically represent the analysis process that was followed, Figure 1 shows a flowchart of the essence of this process.

### 2.1. Study Area and Data Source

As already mentioned in Section 1, the data to be analyzed were those of the PM${}_{2.5}$ concentration in downtown Quito during the six-year period from 2015–2020, taken from hour to hour. In short, in this research, we propose to analyze the historical data collected by the Quito Environmental Protection Agency, and the FH62C14 continuous ambient particulate monitor was used to carry out the measurements. In addition, the methodology used by the Quito Environmental Protection Agency to carry out PM${}_{2.5}$ concentration measurements and store the results of said measurements is explained at http://www.quitoambiente.gob.ec/index.php/informes (accessed on 30 September 2022).

### 2.2. Initial Data Analysis

To begin the study of the data, a preliminary analysis of the available observations was made [1,2,59,60,61,62]. This study was carried out through an exploratory analysis of the data. Here, graphs and numerical characteristics were used to describe the data. This allowed the researchers to be able to interpret the data and notice both the presence of missing observations and observations incompatible with reality.

In addition, a study of the possible differences that could exist between the meteorological conditions in 2019 and 2020 was carried out. This allowed demonstrating whether changes in the meteorological conditions, during these years, could have influenced the PM${}_{2.5}$ concentration.

The initial data analysis showed that there was a large amount of missing data. Therefore, this research attempted to fill in some missing data. In order to do this, the observations from the preceding five years were considered (i.e., the data collected since 2010 were used), and the following recognition stood out:

1. There were observations with zero values.
2. There were observations with negative values.
3. There were missing observations that were isolated.
4. There were consecutive sequences of hours, even days, where there were no measurements at all.

Therefore, to solve these difficulties and with the aim of modifying the data, which were handled as little as possible, it was decided to do the following:

1. Given the incompatibility with the reality that there were observations with either null or negative values, it was decided to delete all of them.
2. It was decided to not fill in any of the missing observations of the year 2020. This was done to avoid any change in the measurement results of 2020, because this year was to be compared with the others.
3. February 29 was deleted from the two leap years included in the study.

In accordance with the above, information about the data that each of the years under study should have is shown in Table 1. From this table, it can be concluded that, for the analysis, the number of observations that were assumed to be missing between 2015 and 2019 was 2212. This represents slightly more than 5% of the total number of values. Of course, those missing observations of each year did not occur at the same time instants.

### 2.3. Missing Data

Due to the fact that there were missing data in the historical series, this lack of data implied a lack of information, which, in long series, can lead to perceptual changes in the subsequent analysis of the data. Therefore, it was necessary to include some missing data from the time series under study.

According to classical texts on missing data [63], missing data in a univariate time series can be considered as either the loss of data due to chance (i.e., missing at random (MAR) or missing completely at random (MCAR)) or the loss of data that are not randomly missing (i.e., missing not at random (NMAR)). In our case, the non-observation of some values could have occurred randomly, but it can be ensured that this loss has not happened randomly when there are several consecutive hours without data. This could be due to the sensors having stopped measuring, because they broke, or were under repair, or the power went out, or due to other reasons that depend clearly on human beings and not on nature.

Within the wide variety of methods that exist to fill in missing data from a time series, one of the objectives of this research was to fill in some of the missing data in the PM${}_{2.5}$ concentration between 2015 and 2019 using two ways: (1) interpolation and (2) classic and robust statistics [64] of the data prior to the missing observation relative to the five previous years.

In short, what was intended was to obtain 4096 observations in each semester of each year, with the intention that the same days and hours of each year can be counted, and not to fill in any data for the year 2020.

In order to do the above, first of all, classical and robust estimators were used with the data from the five years prior to the missing observation. Therefore, all available data from January 2010 were used. That said, if one or more of the data from 2015 were missing, then the data from the years 2010 to 2015 were used. Second, well-known methods of interpolation by polynomials with different degrees, piecewise linear interpolation, interpolations by near values, splines, the Akima method, and Fourier series were also used [65,66,67].

Here, to verify the effectiveness of the interpolation methods, the data for the month of March 2019 were analyzed, because 2019 was one of the last years under study, and also, this month only had one missing observation. Given a missing observation, *m*, taking into account its year, month, day, and time and the previous five years, 35 estimates of said missing observation were found.

The location estimators used in the research were the mean, as a classical estimator, and the following four robust estimators [64,68,69]: median (Me), Andrew’s wave ($T_{wa}\left(c\right)$ for *c* = 2.4π), and α-trimmed mean (T(α) for α = 0.1 and α = 0.2). Of course, other robust estimators could have been chosen, but based on what has been shown in other studies carried out by the authors of this paper [59,60], the robust estimators chosen in this research are the ones that show the least analogies with each other when they are used to analyze air pollution data.

The difference between the estimates is in the dataset to which they are applied. In this sense, in this research, 8 sets were built to fill in the data. These sets were the ones that were analyzed using two different forms of verification: (1) interpolation methods and (2) classical and robust statistics.

At this point, it is important to mention that the above was performed to find the best method of filling in the data. Therefore, in the end, as the case may be, the best form, method, and statistic were proposed.

As a last comment on the above, the 8 sets mentioned above were not all disjoint two by two, because it was sought to take maximum advantage of the redundancy in the data analysis. From our point of view, this redundancy is good because, for the case under study, it brings out the intrinsic characteristics of the data.

The 8 sets were as follows:

- Set 1: This set was made up of data from the 15 days before and 15 days after the same year of data *m*. On each of those days, three hours before, three after, and the hour of *m* were used. In addition, the 3 h before and after the missing data were used. Thus, this dataset, with around 3 h, has 216 observations, and it was called “the month of the missing observation”. For this dataset, 5 estimators were found: (1) mean, (2) median, which was called the general estimate (GE), (3) $T_{wa}\left(2.4\pi \right)$, (4) T(0.1), and (5) T(0.2).
- Set 2: This dataset was built by using the observations of the 3 days before, the 3 days after, and the day of data *m*. On each of these days, 3 h before, 3 h after, and the hour of *m* were used in the 5 years prior to the year of observation *m*. In addition, the above was performed together with the data of the 3 days before and 3 days after the same year of *m* of the 3 h before, the 3 after, and the 3 h before and after the hour of *m*. The amount of data in this set was equal to 293 observations. This set was called “data for one week in a three-hour frame over five years”. Finally, for these observations, the five estimators mentioned above were also found.
- Set 3: The five estimators mentioned above were also applied to this dataset. This dataset was analogous to Set 2, but only with the data for the day before and the day after the date of *m* in the previous five years. In total, the amount of data in this set was equal to 76 observations. This set was called “data for one day with a three-hour frame for five years”.
- Set 4: This dataset consisted of the 3 h before, the 3 h after, and the same time of *m* of the previous 5 years and of the year itself, except, of course, for the time of the missing data. In total, the amount of data in this set was equal to 41 observations. Finally, for these observations, the five estimators mentioned above were also found.
- Set 5: This dataset was built by choosing only 1 h before, 1 h after, and the same hour of *m* of the previous 5 years and the hour before and after of *m*. The amount of data in this set was equal to 17 observations, and the five estimators mentioned above were also found.
- Set 6: This dataset was analogous to the previous one, but did not include the time before and after *m*. Therefore, the five estimates of 15 observations were obtained.
- Set 7: This dataset was built by using data from the same day and time in the previous five years. Therefore, as this set consisted of only 5 observations, only the following estimators were used: mean, median, $T_{wa}\left(2.4\pi \right)$, and T(0.2). However, in the event that all the data were missing, the GE estimate was chosen, because this estimate was the one that gave the best results when evaluating the fit models from among the five estimators. In this case, the number of observations considered was equal to 216, which were made up of data from the 15 days before and after the missing data and 3 h before and after the missing data.
- Set 8: This dataset was built by using the data before and after the missing data, and the mean of both values was used as an estimator, that is a linear interpolation, unless one of them was missing, in which case the GE estimate was chosen.

Once the previous sets had been built, the next step was to select the most appropriate method to complete the data, testing with a good reference month. In this sense, the month of March 2019 was taken as a reference, because this month only lacked one observation. Then, in order to select the method, 100 pieces of data from this month were randomly removed. Since the data loss mechanism was NMAR, the March 2019 data wee transformed using data from other months that had different missing observations.

At this point, it is important to mention that, later on, it was seen that several criteria were used to be able to say if the proposed method was good. However, some criteria highlighted things that others did not. Therefore, it is difficult to say with 100% confidence that one criterion is much better than another. Therefore, in the end, after having tested various criteria for comparison, the most suitable criteria were chosen, perhaps for reasons of convenience, and those criteria were taken as the basis to support what was done.

The months chosen to carry out this analysis were: (1) August 2013, which had 111 missing observations, (2) February 2010, which had 64 missing observations, (3) September 2020, which had 49 missing observations, and (4) August 2010, which had 33 missing observations.

After modifying the data, the estimators and interpolations considered were applied. The filled in data were then compared with the true data, using Pearson’s linear correlation coefficient (*r*), Kendall’s coefficient (τ), Spearman’s rank correlation coefficient (ρ), and the residual sum of squares (RSS) [70].

### 2.4. Construction of Feature Vectors

The information collected during the measurement process was diverse, because it was made up of measurements of the same variable under study, but with characteristics that could be different. Therefore, this information did not have a specific distribution in terms of forms and repetitions. Moreover, when analyzing the measurements, large homogeneous areas were appreciated in relation to other small ones that had, for example, very high values and borders between them. This is the reason why the elements that made up the data sequences could be forming configurations that depend on the scale. Consequently, it was possible to identify some structures on a small scale, because they had little contrast between them, while there were other structures that were better appreciated at larger scales.

The procedures that are presented in this research were used to classify the 6 years of measurements that were considered. In addition, these procedures compared all the data corresponding to the years 2015–2019 with the data corresponding to the year 2020, because, due to the COVID-19 pandemic, it was in 2020 that very noteworthy changes in life began to occur in the cities.

That said, knowing that there are many possibilities offered by PM${}_{2.5}$ concentration measurements in cities, it is true that a small fraction of the measurements could contain enough information to classify them. Therefore, this allowed carrying out the study of the possible characteristics of the measurements.

In this research, the feature vectors were obtained by using a wavelet transform, which allows relevant information to be highlighted. Due to the repeated information contained in the data sequences, the statistical characteristics in different areas were highly variable. On the other hand, when representing the sequences at different scales, using the wavelet transform, the local statistical characteristics were more stable and constant everywhere.

Here, the size of the sequences was adapted to be able to build a non-overlapping partition of sequences of size $2^{k}$, and for each of these cells, a multiscale decomposition of the discrete Daubechies wavelet family with 5 levels was performed [37]. In this paper, the 6 years considered for the analysis had $2^{12}=$ 4096 observations in each semester. Thus, to carry out the comparative study of the years, each sequence of size $2^{13}=$ 8192 was divided into two parts: one with the even observations and the other with the odd ones. Then, each of these parts was divided into two sequences of $2^{11}=$ 2048 elements each. Therefore, in each year, there were 4 subsequences (i.e., 4 trimesters) of size 2048 elements.

A Daubechies wavelet transformation with *N* = 5 levels was applied to each of these subsequences. Then, on the one hand, $A_{i}$, for *i* = 1,…,5, was obtained, containing the approximation coefficients, and, on the other hand, $D_{i}$ was obtained, containing the small-scale detail coefficients.

Finally, a statistical analysis of the decomposition of each of the sequences was carried out. Said analysis was performed both with statistics of the marginal distributions and with the joint statistics of the decomposition of each of the 4 subsequences, obtaining a feature vector in a 320-dimensional vector space.

### 2.5. Wavelet Coefficients

Wavelet transforms are orthonormal basis decompositions of vector spaces. However, there may be significant dependencies between scales. Although there may be distortions in the calculations, due to quantization errors, what happens is that the characteristic structures of the sequence tend to have coefficients substantially different from zero on various scales. Concluding that, given a fixed wavelet coefficient of a scale, there were dependencies in adjacent orientations on higher-level scales. Furthermore, the statistical characteristics of the marginal decompositions were insufficient to adequately capture the underlying structure of the given sequence. For all this, in this research, central moments of up to the fourth order were proposed to capture these dependencies.

Here, to show the dependence between the wavelet coefficients, the coefficients of the next level were doubled and the coefficients of two more levels were quadrupled. This was performed so that all three sequences were the same length. Next, to find the estimate of each feature vector, a one-level step-by-step regression was performed with the next two levels. Thus, once the estimate was obtained, the true value was compared with the estimate using the quotient, and the logarithm to the base 2 of the quotient was considered [43].

In the case under study, to characterize the time series by year, semester, and trimester of the same size, a vector that gathered all the characteristics of each subsequence was considered, and the following statistics were taken into account [43]:

- The mean, standard deviation, skewness, kurtosis, minimum, maximum, and 2nd and 98th centiles of the entire subsequence, of the detail coefficients of the N=5 levels and the approximation coefficients of the fifth level.
- To try to partially capture the dependencies between scale neighborhoods, it is proposed that, given a coefficient of the scale of level *k*, with k=1, 2 or 3, its value be predicted using the multiple regression method with least squares. Then, the duplicated coefficients of the k+1 level and the quadrupled coefficients of the k+2 level were established as the predictors.
  The values of the coefficients $D_{k}$ were put in columns, and the rows of the matrix *Q* consisted of the 2 predictors $D_{k+1}$ and $D_{k+2}$. Therefore, the matrix *Q* had dimension $2^{\left(11-k\right)}$ rows and 2 columns. The least-squares estimate, ${\hat{D}}_{k}$, of $D_{K}$ is given by Equation (1):
  (1) $${\hat{D}}_{k}=Q\cdot {\left(Q^{t}\cdot Q\right)}^{-1}\cdot Q^{t}\cdot D_{k}$$
  where $Q^{t}$ is the transpose of *Q*.
  Once the estimate was obtained, the estimation error was found by Equation (2):
  (2) $$E_{k}=\log_{2}\left(\right|D_{k}\left|\right)-\log_{2}\left(\right|{\hat{D}}_{k}\left|\right)$$
  Finally, the considered statistics were the same as those used previously.

### 2.6. Classification of PM${}_{2.5}$ Concentration by Separation Measures between Feature Vectors

What was said above made it clear that the sequences representing the years, whose length was $2^{13}=$ 8192, were represented by feature vectors of dimension 320. However, from the point of view of the authors of this paper, these vectors are still in a large vector space. Therefore, in order to be able to make a decision about the PM${}_{2.5}$ concentration measurements in the studied area, during the years 2015–2020, the measurements of each measurement point (i.e., of each year) were projected on the same plane in k<p dimensions, where p=320. Furthermore, to carry out the above, the dissimilarities between the feature vectors were analyzed by using the PCA and MDS techniques.

By having *p* variables collected on the *n* analyzed units (i.e., years, semesters, or trimesters), the *p* collected variables were required to reproduce the total variability of the system, and sometimes, most of that variability can be found in a small number, k<p, of principal components. The *k* principal components can replace the initial *p* variables, whereby the original dataset, consisting of *n* analyzed units of *p* variables, was reduced to *n* measures of *k* principal components.

With the application of the PCA technique, a new set of orthogonal axes was found in which the variance of the data was maximized. In addition to providing a solution to the problem of dimensionality reduction, once the transformation had been carried out, it could facilitate its interpretation through the relationships between the variables that were not obvious at first glance [38,39].

Once the feature vectors were built, the comparison between them was also carried out by using the MDS technique. With this technique, underlying discrepancies in similarity judgments were detected, analyzing these discrepancies in terms of distances and the lack of similarity [40,71].

In summary, from a data matrix $X\in M_{n\times p}$, where the rows represent the observations and the columns the variables, the objective was to find the principal coordinates in such a way that they distorted the pairs of distances between the observations as little as possible.

The MDS technique is related to the PCA technique, since they share working methods and, in the case of the Euclidean distances, results. There are two methods: (1) the classic MDS, which starts with a distance matrix, and (2) the nonmetric MDS, which starts from a similarity matrix, D. In either case, the similarity matrix is considered, $L=-\frac{1}{2}D^{2}$. Next, the eigenvalues and eigenvectors of L are found in order to obtain the singular-value decomposition $L=HBH^{t}$, where $H\in M_{n\times p}$ contains the nonzero eigenvectors of L, $B\in M_{p\times p}$ is the diagonal matrix with the eigenvalues of L, and the superscript *t* means the transpose of the matrix. Therefore, L can be expressed as follows: $L=HBH^{t}=HB^{1/2}B^{1/2}H^{t}=\left(HB^{1/2}\right){\left(HB^{1/2}\right)}^{t}\Rightarrow L=YY^{t},\;\mathrm{where}\;Y=HB^{1/2}\in M_{n\times p}$. In this way, by choosing Y, a set of variables is obtained that collect the similarities between the distances [42].

In this research, the nonmetric MDS was used, in which a relationship between similarities is used, and from it, a distance between the elements is obtained; this distance is compared with the initial one, to finally try to minimize a measure of closeness between similarities, $\delta_{ij}$, and distances. This method, unlike the principal component method, has a criterion for measuring the goodness-of-fit, called stress [72]. In this paper, in agreement with other authors [72], the stress is given by Equation (3).

(3) $$S={\left(\frac{\sum \sum_{i<j}{\left(\delta_{ij}-{\hat{d}}_{ij}\right)}^{2}}{\sum \sum_{i<j}\delta_{ij}^{2}}\right)}^{1/2}$$

where $\delta_{ij}$ are the elements of $L=YY^{t}$ and ${\hat{d}}_{ij}$ are the estimated values of the distance between variables (i.e., ${\hat{d}}_{ij}$ are the optimal observed values, according to the nonmetric MDS, which minimizes the stress Equation (3).

When the coordinates represent measurements subject to random fluctuations, it is desirable to assign weights to the coordinates depending on the variability of the measurements. This suggests using a measure of distance that is different from the Euclidean. Therefore, some authors have proposed the Hausdorff distance [71] as a metric to determine the degree of similarity between two objects.

## 3. Results and Discussion

### 3.1. Preliminary Data Analysis

The variables under analysis were as follows:

- $X_{k}$, k=1…6, is the PM${}_{2.5}$ concentration each year: 2015 (k=1), 2016 (k=2), *…*, and 2020 (k=6).
- $Y_{k}$, k=1…12, is the PM${}_{2.5}$ concentration each month: January (k=1), February (k=2), *…*, and December (k=12).
- $Z_{k}$, k=1…7, is the PM${}_{2.5}$ concentration each day: Monday (k=1), Tuesday (k=2), *…*, and Sunday (k=7).
- $W_{k}$, k=1…12, is the PM${}_{2.5}$ concentration pooled for each group of two hours. Specifically, the concentration at 0:00 and 1:00 is represented by $W_{1}$, the concentration at 2:00 and 3:00 is represented by $W_{2}$, *…*, and the concentration at 22:00 and 23:00 is represented by $W_{12}$.

Table 2 shows a statistical summary of the PM${}_{2.5}$ concentration measurements (in $\mu {g/m}^{3}$) taken from downtown Quito each year, from 2015 to 2020. In this research, outliers were considered to be those values that were above the third quartile plus 1.5-times the interquartile range [73]. The last column of Table 2 represents the percentage of outliers that each variable had.

As seen in other studies on PM${}_{2.5}$ concentration [1], the summary statistics (Table 2) showed both that the mean was greater than the median and that the skewness as greater than zero for all variables. In addition, all the kurtosis values were greater than 6, becoming greater than 1000 in the year 2017. All this indicates either that the variables follow a heavy-tailed distribution [64,74] or that this behavior is due to the presence of a mixture of distributions.

**Figure 2:**
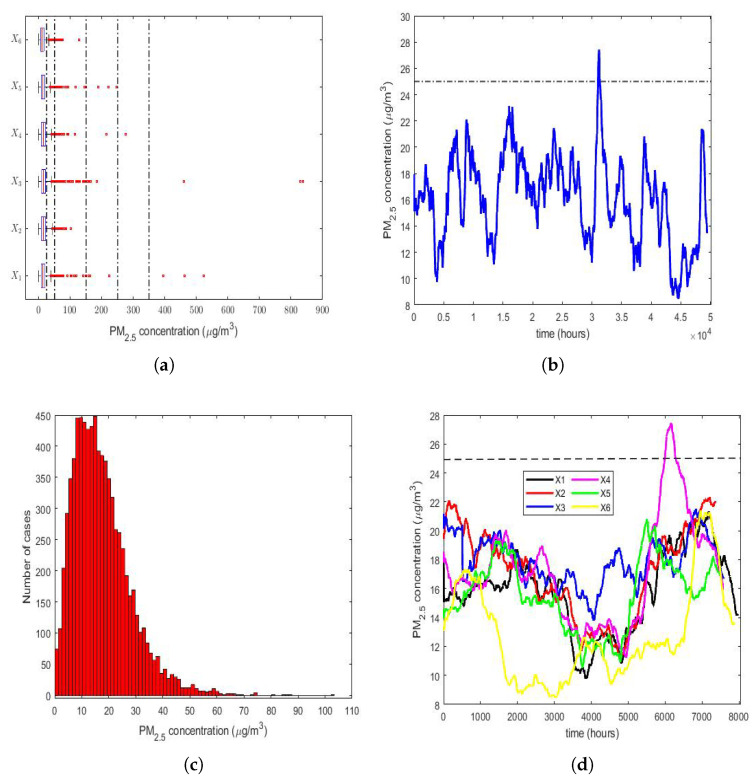
Box plot of each variable, moving average of the time series consisting of all observations, histogram of the year 2016, and moving average of each variable. The outliers are represented by red circles, and the dashed lines (5 vertical dashed lines in (**a**) and 1 horizontal dashed line in (**b**,**d**)) indicate intervals of air pollution levels: desirable level [0, 25 µg/m^3^), acceptable level [25 µg/m^3^, 50 µg/m^3^), caution level [50 µg/m^3^, 150 µg/m^3^), alert level [150 µg/m^3^, 250 µg/m^3^), alarm level [250 µg/m^3^, µg/m^3^), and emergency level [350 µg/m^3^, *∞*). (**a**) Multiple box plots: $X_{1}$ (2015), *…*, $X_{6}$ (2020). (**b**) Moving average of all observations from 1 January 2015 to 31 December 2020. (**c**) Histogram of the year 2016 ($X_{2}$). (**d**) Moving average of each variable: $X_{1}$ (2015), *…*, $X_{6}$ (2020).

The four graphs shown in Figure 2 are as follows:

- A box plot diagram of all years (2015–2020) (Figure 2a).
- The moving average (MA) of the time series consisting of all observations from 1 January 2015 to 31 December 2020 (Figure 2b).
- A histogram of the year 2016 (Figure 2c), because $X_{2}$ is the variable that has the lowest skewness and the lowest kurtosis (see Table 2).
- The MAs of all years (2015–2020) (Figure 2d).

The size that was used to carry out the MA smoothing was equal to 720, because this is the number of PM${}_{2.5}$ concentration measurements taken in a 30-day month. However, it should not be forgotten that not all possible data were available, because there were missing data.

According to what is indicated in the Quito air quality reports that were issued by the Quito Environmental Protection Agency from 2003 to 2019 (these reports are available online for the general public and can be downloaded from http://www.quitoambiente.gob.ec/index.php/informes (accessed on 30 September 2022)), the air pollution categories due to the average PM${}_{2.5}$ concentration in Quito over 24 h are as follows:

- Desirable level: [0, 25 µg/m^3^).
- Acceptable level: [25 µg/m^3^, 50 µg/m^3^).
- Caution level: [50 µg/m^3^, 150 µg/m^3^).
- Alert level: [150 µg/m^3^, 250 µg/m^3^).
- Alarm level: [250 µg/m^3^, 350 µg/m^3^).
- Emergency level: [350 µg/m^3^, *∞*).

Therefore, in order to locate the measured values of the PM${}_{2.5}$ concentration levels in the above-mentioned 6 air pollution categories, five vertical dashed lines are drawn in Figure 2a to separate each of these 6 categories, indicating the air pollution level. From Figure 2a, every year had PM${}_{2.5}$ concentration outliers that were above the acceptable level; 4 years had outliers that were above the caution level, although they were very few; 3 years had either 1, 2, or 3 outliers above the alert level; 2 years had either 2 or 3 outliers above the alarm level, which were within the interval corresponding to the emergency level. Although the percentage of outliers of each year was approximately less than or equal to 3% (see Table 2), the above indicates that the observations did not follow a Gaussian distribution.

Figure 2b indicates that the PM${}_{2.5}$ concentration was stable during the time period analyzed. Moreover, it was observed that, by smoothing the observations, the desirable level of air pollution was only exceeded on one occasion. In this figure, the upper limit of this level is represented by a dashed horizontal line. Lastly, it can be concluded that exceeding the desirable level of air pollution occurred only at specific moments, and never in a sustainable way.

In general, Figure 2d shows that the time series of the PM${}_{2.5}$ concentration values of the year 2020 ($X_{6}$) had lower values than the other time series (i.e., $X_{1},\;\ldots \;X_{5}$), but the graphs appear interlaced among the time series of each of the first 5 years of the study.

**Figure 3:**
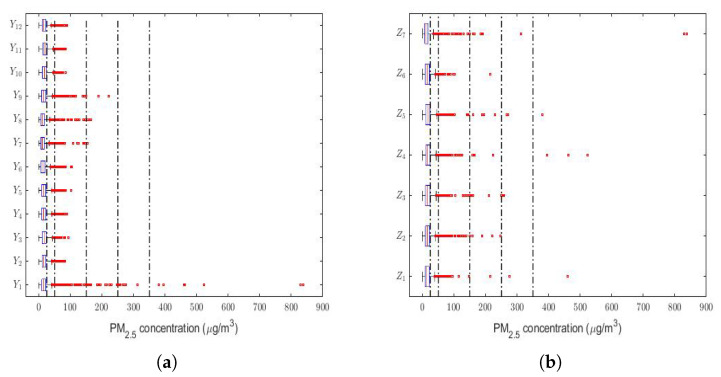

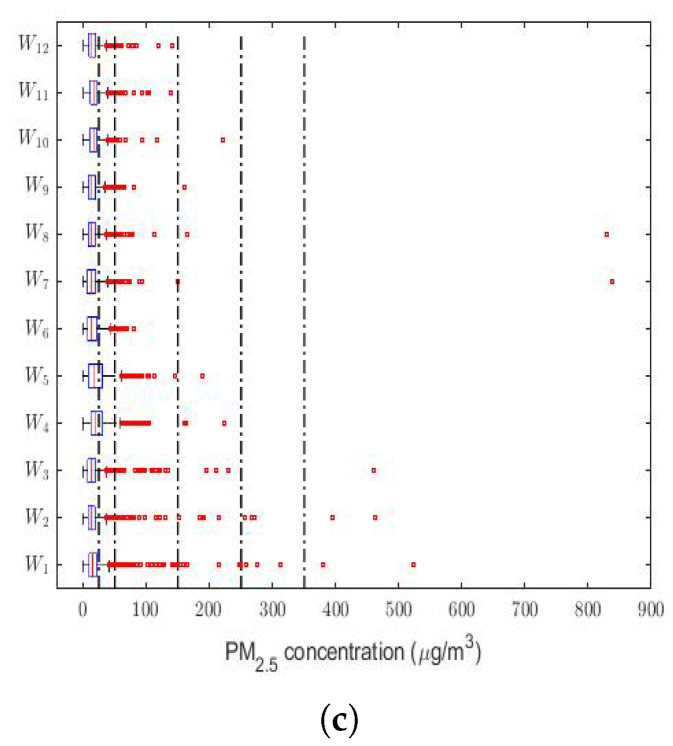
Multiple box plots for months, days, and day hours every two hours. The outliers are represented by red circles, and the 5 vertical dashed lines in each figure indicate the intervals of air pollution levels: desirable level [0, 25 µg/m^3^), acceptable level [25 µg/m^3^, 50 µg/m^3^), caution level [50 µg/m^3^, 150 µg/m^3^), alert level [150 µg/m^3^, 250 µg/m^3^), alarm level [250 µg/m^3^, 350 µg/m^3^), and emergency level [350 µg/m^3^, *∞*). (**a**) Box plots of the months: January ($Y_{1}$), *…*, December ($Y_{12}$). (**b**) Box plots of the days: Monday ($Z_{1}$), *…*, Sunday ($Z_{7}$). (**c**) Box plot of the day hours every two hours: 0:00–1:00 ($W_{1}$), *…*, 22:00–23:00 ($W_{12}$).

Finally, a box plot of the observations for months, days of the week, and every two hours of the day is provided in Figure 3. This figure shows that all the variables had extreme observations on the right, which were generally close to each other, and that in very few cases, some of these observations fell within the emergency level of air pollution due to the PM${}_{2.5}$ concentration.

Most of the abnormally high observations occurred in the month of January (see Figure 3a) and in the early and middle hours of the day (see Figure 3c). Without taking the abnormally high observations into account, the behavior of the variables was very similar between all the months (see Figure 3a), the days of the week (see Figure 3b), and the hours of the day (see Figure 3c). Furthermore, Figure 3 shows that a large number of observations were at the caution level, which were outliers. Hence, this seems to indicate that these variables followed heavy-tailed distributions.

### 3.2. Results Taking into Account the Possible Impact of Meteorological Factors on the PM${}_{2.5}$ Concentration Levels

The analysis of the role that meteorological factors play in the PM${}_{2.5}$ concentration levels is of paramount importance [7,75,76,77,78], because changes in these factors can considerably affect the concentration levels of this air pollutant. Therefore, the aim of the next paragraphs of the paper is to make a comparison between different meteorological variables that could influence the PM${}_{2.5}$ concentration levels in the region under study. To make this comparison, we focused on the years 2020 and 2019, which was the year just before the pandemic.

#### 3.2.1. Descriptive Statistics: 2020 vs. 2019

The meteorological variables that were taken into consideration were as follows:

1. Wind speed (WS).
2. Relative humidity (RH).
3. Rainfall (R).
4. Solar irradiance (SI).
5. Wind direction (WD).
6. Temperature (T).
7. Atmospheric pressure (AP).
8. Ultraviolet index (UV).

**Figure 4:**
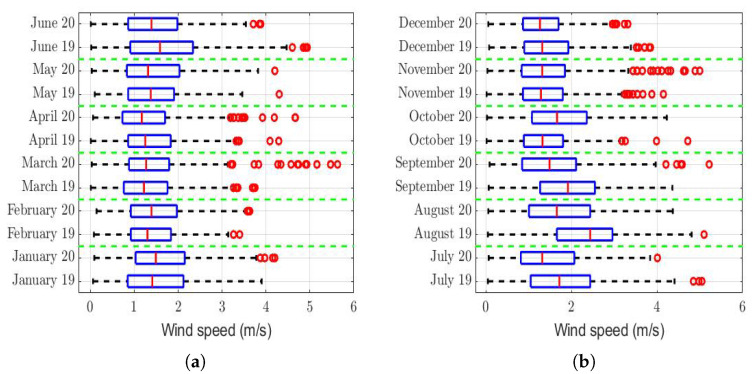
Box plot of the wind speed (m/s): 2019 vs. 2020. The red circles represent the outliers. (**a**) First semester. (**b**) Second semester.

**Figure 5:**
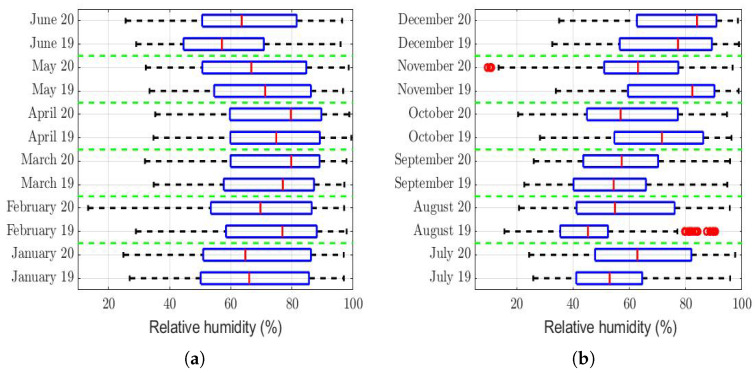
Box plot of the relative humidity (%): 2019 vs. 2020. The red circles represent the outliers. (**a**) First semester. (**b**) Second semester.

**Figure 6:**
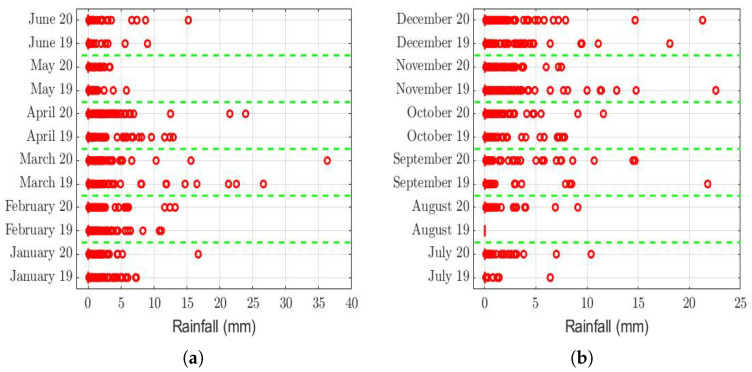
Box plot of the rainfall (mm): 2019 vs. 2020. The red circles represent the outliers. (**a**) First semester. (**b**) Second semester.

**Figure 7:**
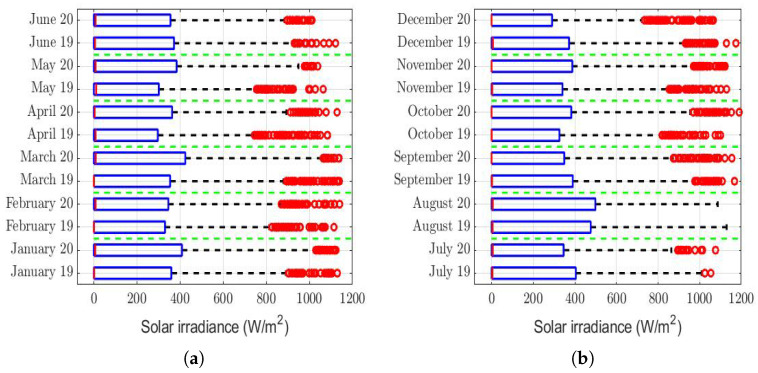
Box plot of the solar irradiance (W/m${}^{2}$): 2019 vs. 2020. The red circles represent the outliers. (**a**) First semester. (**b**) Second semester.

**Figure 8:**
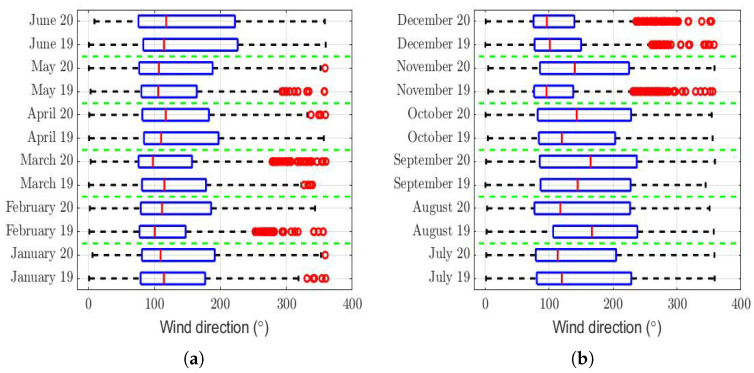
Box plot of the wind direction (°): 2019 vs. 2020. The red circles represent the outliers. (**a**) First semester. (**b**) Second semester.

**Figure 9:**
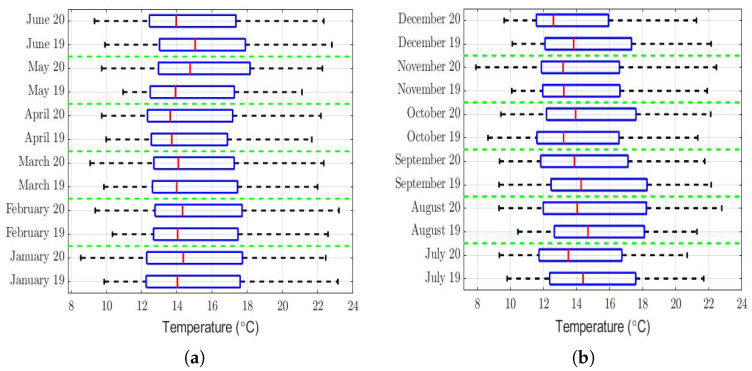
Box plot of the temperature (°C): 2019 vs. 2020. (**a**) First semester. (**b**) Second semester.

**Figure 10:**
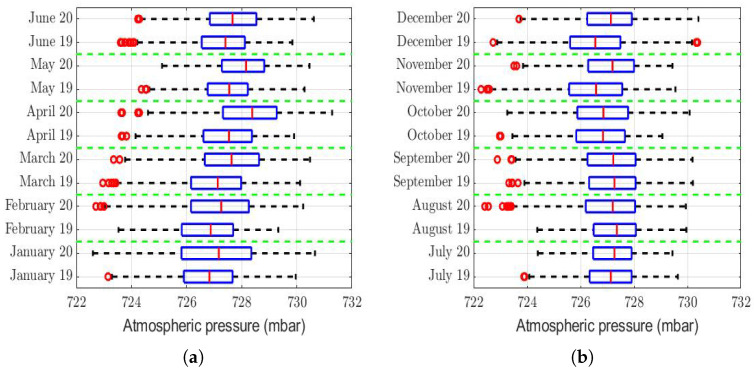
Box plot of the atmospheric pressure (mbar): 2019 vs. 2020. The red circles represent the outliers. (**a**) First semester. (**b**) Second semester.

**Figure 11:**
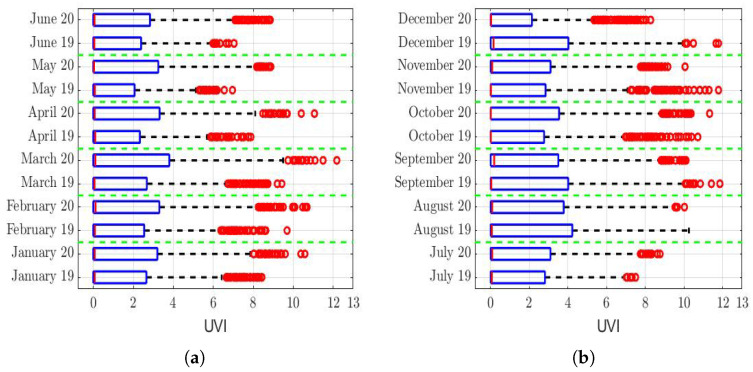
Box plot of the ultraviolet index (UVI): 2019 vs. 2020. The red circles represent the outliers. (**a**) First semester. (**b**) Second semester.

The box plots of the measurements of these meteorological variables are provided in Figure 4, Figure 5, Figure 6, Figure 7, Figure 8, Figure 9, Figure 10 and Figure 11. These figures show that there were no considerable differences in terms of wind speed in 2019 and 2020. In addition, with respect to relative humidity, it can be said that, although there were small differences in the summer months, these were insignificant. In the same way, there was not a big difference in terms of rainfall. Regarding this, it is worth saying that in Quito. it seems that it does not rain in large quantities; in fact, in August 2019, it did not rain at all, according to the measurements considered.

Furthermore, as for the previous meteorological variables, it seems that the box plots indicate that there were no considerable differences in terms of solar irradiance, wind speed, temperature, and atmospheric pressure.

Finally, it cannot be said that the ultraviolet index in 2019 was considerably different from that of 2020.

#### 3.2.2. Comparison by Means of Statistical Hypothesis Testing: 2020 vs. 2019

In this part of the paper, the possible differences between the meteorological variables are established, in the two years under study, using statistical hypothesis testing of homogeneity between populations.

That said, in this paper, it was decided to establish nonparametric hypothesis testing due to the following reasons: (1) in view of the box plots (shown in Figure 4, Figure 5, Figure 6, Figure 7, Figure 8, Figure 9, Figure 10 and Figure 11), it does not seem that the distributions of the considered meteorological variables followed a normal distribution, and (2) independence between observations of the same meteorological variable cannot be guaranteed, because the value of each observation was reasonably dependent on the value of the previous observation.

Through statistical hypothesis testing, it was intended to analyze both differences between values and their respective medians and the size of these differences. The above was carried out using the Wilcoxon rank-sum test [70].

Given a meteorological variable of a specific month, the null hypothesis ($H_{0}$) was that the distribution of that variable in 2020 was equal to the distribution of the same variable in 2019 plus *K*, *K* ∈ R. The alternative hypothesis ($H_{1}$) was that the distribution of that variable in 2020 was different from the distribution of the same variable in 2019 plus *K*.

The results of the test are shown in Table 3. If in this table, any of the rows indicating the meteorological variable name shows the value 0 in the month columns, this means that $H_{0}$ was not rejected. On the contrary, if the value is 1, then $H_{0}$ was rejected in favor of $H_{1}$. In addition, the values that appear in the rows called *K* are the ones that must be entered in order not to reject $H_{0}$, at the α=0.05 significance level. Finally, the rows called *p*-value [70] show the probability that the test statistic had a value that was equal to or greater than the calculated value from the data shown in the random sample, assuming $H_{0}$ is true.

Table 3 shows that, in six months, the wind speed was somewhat higher in 2019, while in four months, it was higher in 2020. However, these variations were not systematic. In addition, given that the largest difference was found in August, but this was only 0.6 m/s, it cannot be said that the wind speed had a greater influence on the PM${}_{2.5}$ concentration in one year than in the other, because the range of variability of the observations was between 0 and 6 m/s (see Figure 4).

Table 3 also shows that the difference in relative humidity (see Figure 5) between the months of 2019 and 2020 was at most equal to 10%. Additionally, it shows that there was no behavior pattern that indicated that the relative humidity during the months of a year was always greater than or equal to that in the same months of the other year. In fact, in one year, the summer had the highest relative humidity value, but the autumn of the same year had the lowest value. Therefore, the variations shown in Table 3 do not seem to be large enough to ensure that the relative humidity had significantly more influence in one year than in the other.

Considering the instants in which the rainfall was different from 0, both in 2019 and in 2020, Table 3 shows that it was the same in both years, with the exception that in August 2019, it did not rain. In addition, the difference of 0.2 mm (i.e., *K*=0.2 mm) in the months of October 2019 and 2020 was insignificant compared to the range of values that this variable took (see Figure 6). Therefore, there are no sufficient arguments to say that the rainfall caused the PM${}_{2.5}$ concentration to be higher in one year than in the other.

Solar irradiance took very large values throughout the day, during each month. This is shown in Figure 7, which shows extreme values of up to 1200 W/m${}^{2}$. Therefore, the rows showing this variable in Table 3 tell us that the homogeneity between the solar irradiance in 2019 and the solar irradiance in 2020 cannot be rejected. Nevertheless, it is true that the null hypothesis ($H_{0}$) was rejected in June, but it is also true that this happened in a very weak way, because $H_{0}$ was accepted at the α = 0.045 significance level. On the other hand, there were differences in the first months of both years, but these were insignificant compared to the values taken by the meteorological variable. Therefore, it cannot be said that the solar irradiance influenced so much as to make the PM${}_{2.5}$ concentration higher in one year than in the other.

However, it is important to mention that as the *p*-value = 0.045<α = 0.05, when comparing the solar radiation of June 2019 with that of June 2022, we modified the test for a linear transformation in which the dependent variable (i.e., *X*) represents the distribution of solar irradiance in June 2020 and the independent variable (i.e., *Y*) represents the distribution of solar irradiance in June 2019. Now, $H_{0}$: X=0.9·Y+0.001, and $H_{1}$: X≠0.9·Y+0.001.

Once the above-mentioned linear transformation was performed, the Wilcoxon rank-sum test was applied, and it was concluded that $H_{0}$ was accepted, with *p*-value = 0.161 > α=0.05. Note that this model showed that the solar irradiance in June 2020 was 10% less than the solar irradiance in June 2019. Therefore, the foregoing allowed us to conclude that there were no notable systematic differences between the distributions of solar irradiance by months, between the years 2019 and 2020, because the differences were mainly due to very low constants.

Regarding the wind direction, the rows of Table 3 of this variable show that the hypothesis that the difference between the wind direction between the months is a constant was not rejected. Furthermore, the maximum value of the difference that is reached in said table was less than 10% of the maximum value that the variable can take (see Figure 8). Moreover, due to the sign of this difference alternates throughout the months of the year, this could indicate that the differences were not systematic and that the influence of this variable may not have been significant.

Regarding the temperature, the rows of Table 3 of this variable show that its value was a little lower in the summer months of 2020 than in the summer months of 2019. However, this table also shows that this difference was very small compared to the range of values that this variable took (see Figure 9). Additionally, the runs had values that were either very small or equal to 0. Therefore, there were not enough arguments to say that the temperature in 2019 was significantly different from that of 2020.

Another variable that was analyzed was atmospheric pressure. The rows of Table 3 of this variable show that, in 8 months of the year, this was higher in 2020 than in 2019, although this difference was very small compared to the range of values that this variable took (see Figure 10). Therefore, if this variable had any type of influence on the differences that existed in the PM${}_{2.5}$ concentration in 2020 compared to 2019, this influence was small. In addition, it is noteworthy that most of this difference appeared in the first half of the year, when the lockdowns had either not yet began or just began.

Regarding the ultraviolet (UV) index analysis (see Figure 11), the last rows of Table 3 show that the hypothesis that the UV index during 2019 and 2020 was the same cannot be rejected, except for the month of December. In fact, it cannot be said that, in December, this difference was equal to a constant. In a nutshell, we think this is because all the December 2019 outliers were larger than the December 2020 outliers (see Figure 11b). That said, since this difference only occurred in the last month of the year, this did not materially influence what happened during the previous 11 months of the year. Therefore, the structure of the PM${}_{2.5}$ concentration of all the year 2020 cannot be significantly affected by the fact that the UV index of December 2019 was different from that of December 2020.

The analysis of this last variable deserves special attention, because the sequences of missing values had sizes smaller than those corresponding to one week. However, more than half of the month of December 2020 was missing observations. To verify this, two periods were considered. The first (P1) was from 0:00 on 1 December to 11:00 on 19 December. On the other hand, the second period considered (P2) was from 12:00 on 19 December to 23:00 on 31 December. Table 4 shows the data classified as either missing or not in the month under analysis.

Given the large amount of missing data in period P1 (see Table 4), it was decided to carry out the statistical hypothesis test of the equality of the distributions using only the data from period P2, for which the null hypothesis ($H_{0}$) was that the UV index distribution in P2 of December 2020 was equal to the UV index distribution in P2 of December 2019. The alternative hypothesis ($H_{1}$) was that the UV index distribution in P2 of December 2020 was different from the UV index distribution in P2 of December 2019.

Taking the above into account, the application of the Wilcoxon rank-sum test showed that $H_{0}$ was accepted with *p*-value = 0.0938>α = 0.05. Therefore, the hypothesis that the UV index in 2019 was similar to that of 2020 can no longer be rejected with a 95% confidence level.

The above explanation was important, because it showed that it cannot be said that the behavior of the meteorological variables had a significantly different influence on the PM${}_{2.5}$ concentration in 2019 with respect to the one in 2020 in the study region.

### 3.3. Missing Data Imputation

Taking into account what was explained in Section 2.3, 100 data from March 2019 were randomly removed. By doing this, it was observed that both the missing data were generally isolated observations and at most there were sequences of four missing data. In addition, all the interpolations that were tested gave better results than any of the designed estimates, which indicated that, with runs of few observations, it seems better to interpolate.

**Figure 12:**
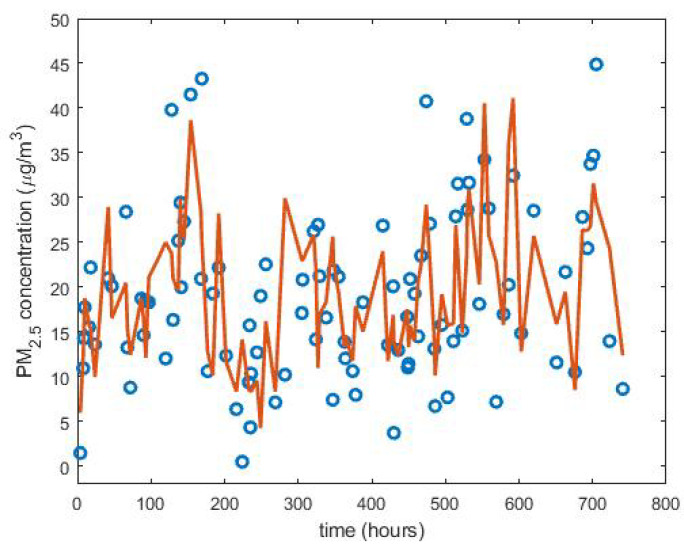
True points for the month of March 2019 (shown with blue circles) and the result of the piecewise interpolation method (shown by the curves in red). Here, 100 data were randomly removed from March 2019, and the piecewise linear interpolation method was the one that gave the best results.

For the case study, the 100 data were eliminated in a uniform way, and the best results, with the four methods to evaluate the model, were obtained by piecewise linear interpolation (see Figure 12). However, Pearson’s linear correlation coefficient was 0.68, which is not very high.

When 111 data from March 2019 were deleted in the same positions as the ones of the 111 missing observations of August 2013, a succession of 82 missing observations was obtained, another of 24 missing observations, and another 5 remaining observations, which were sequences of 1 or 2 missing data. Now, March 2019 was filled in by putting the estimated data in the same positions as the ones of the 111 observations of August 2013 that were missing.

Unlike the previous case, none of the interpolations used were among the best models evaluated. Now, the six best results were obtained with the mean ($Mean^{D}$), Andrew’s wave ($T_{wa}^{D}\left(2.4\pi \right)$) of the dataset for one day over five years, and the mean ($Mean^{W}$), median ($Me^{W}$), Andrew’s wave ($T_{wa}^{W}\left(2.4\pi \right)$), 0.1-trimmed mean ($T^{W}\left(0.1\right)$), and 0.2-trimmed mean ($T^{W}\left(0.2\right)$) of the dataset for one week over five years. Table 5 shows the statistics that were used to evaluate the models. In view of these results, in this case, it was decided to choose $Mean^{D}$ for filling in the suppressed data.

When 64 data located in the same positions of the 64 missing observations of February 2010 were removed from March 2019, a succession of 47 missing data was obtained along with other of 5 missing data and another of 4 missing data. The rest were missing data located in isolated positions. Now, the best results were obtained with four robust estimates: the GE estimate and Andrew’s wave ($T_{wa}^{M}\left(2.4\pi \right)$) of the data for the month of the missing observation and the median ($Me^{5}$) and Andrew’s wave ($T_{wa}^{5}\left(2.4\pi \right)$) of the data corresponding to the missing observation in the previous five years.

Table 6 shows the statistics that were used to evaluate the models, and in view of the results, it was decided to select $Me^{5}$ for filling in the suppressed data. Furthermore, the values obtained in this way were highly correlated with those obtained with $T_{wa}^{5}\left(2.4\pi \right)$, because $r_{Me^{5},T_{wa}^{5}\left(2.4\pi \right)}=0.961$.

When 49 data located in the same positions as the ones of the 49 missing observations of September 2020 were removed from March 2019, two successions of 12 missing data were obtained along with another succession of 11 missing data, another of 8 missing data, one more of 4 missing data, and finally, 2 missing data that were located in isolated positions.

In this case, based on the methods used to evaluate the models, the best results were also obtained by piecewise linear interpolation, although similar results were also obtained with the nearest values and cubic interpolations. Table 7 shows the interpolation methods that were used to evaluate the models.

Lastly, when 33 data from March 2019 that were located in the same positions as the ones of the 33 missing data from August 2010 were deleted, it was observed that these missing data formed a single succession of length 33. Now, with the statistics to evaluate the models, it was obtained that the best results were with estimates, although with very low values of the correlation coefficients. In this case, the best results were obtained with $Me^{D}$ (see Table 8), which is the median of the data for a day with around three hours for five years. Table 8 shows the statistics that were used to evaluate the models. In this table, GE is the general estimate, $Mean^{D}$ is the mean of the data for a day with around three hours for five years, and $T^{D}\left(0.2\right)$ is the 0.2-trimmed mean for the data for a day with around three hours for five years. Additionally, $Mean^{W}$ is the mean for the data for a week with around three hours for five years.

Once all of the above had been performed, the next step was to decide which were the most appropriate methods to fill in the missing observations, based on the characteristics of the sequences of such missing observations. Therefore, for all the above, it was decided to fill in the missing observation sequences using piecewise linear interpolation when the length of the missing observation sequences was less than 20. Likewise, in the case that the length of the sequences of missing observations was greater than 20 and less than 40, the median of the data was chosen for a day with around three hours during five years. This is represented by Set 3. Furthermore, the median of the missing observation in the previous five years was chosen to analyze sequences of missing observations whose length was greater than 40 and less than 70. This is represented by Set 7. Finally, when the length of the missing data sequences was greater than 70, it was decided to analyze the data using the mean of the data for one day with a three-hour frame over five years. This is represented by Set 3.

For the convenience of analyzing the information with missing data, here, it was decided to group the information from each of the semesters into data sequences of a length equal to 4096. Consequently, from the 1059 missing values in the first semester, as indicated in Table 1, it was decided to fill in only 838 to guarantee that the semesters were made up of 4096 data. In the same way, of the 1153 missing values in the second semester, as shown in Table 1, it was decided to fill in only 806 to guarantee that the semesters were made up of 4096 data.

To end this subsection, it is highlighted that what was performed was to homogenize the data for the 5 years prior to 2020. To do this, the empty spaces corresponding to missing observations were filled in with plausible data. Of course, the researchers could also carry out the analysis of the information only with the available data, but this could imply that the result of the study carried out was not the best possible, because the available data were not homogenized.

### 3.4. Description of Feature Vectors and Comparison between Them

Here, the size of the sequences was adapted to be able to build a non-overlapping partition of sequences of size $2^{k}$, and for each of these cells, a multiscale decomposition of the discrete Daubechies wavelet family with five levels was performed. In this paper, the 6 years considered for the analysis had $2^{12}=4096$ observations in each semester. Thus, to carry out the comparative study of the years, each sequence of size $2^{13}=8192$ was divided into two parts: one with the even observations and the other with the odd ones. Then, each of these parts was divided into two sequences of $2^{11}=2048$ elements each. Therefore, in each year, there were four subsequences (i.e., four trimesters) of size 2048 elements.

**Figure 13:**
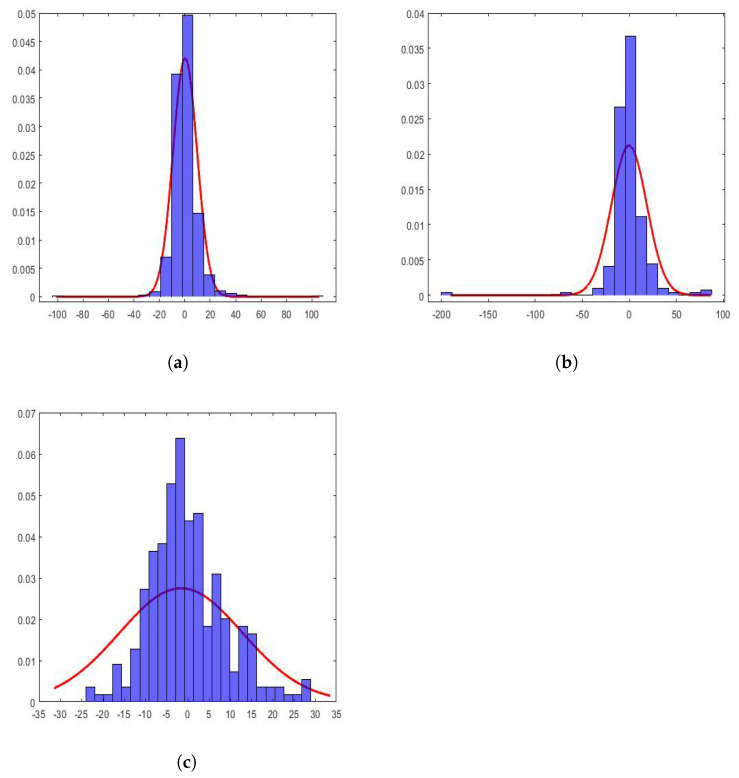
Histograms and wavelet coefficient fits to Gaussian distributions: (**a**) 2015, Level 1, Subsequence 1; (**b**) 2017, Level 3, Subsequence 2; (**c**) 2018, Level 5, Subsequence 3.

In the case under study, the values of the coefficients of the wavelet scales of the considered subsequences followed distributions that were very far from the Gaussian ones. This can be seen in Figure 13. In this figure, the histograms of the following are shown: (1) approximation of the first level, first subsequence of the year 2015, (2) approximation of the third level, second subsequence of the year 2017, and (3) approximation of the fifth level, third subsequence of the year 2019. Additionally, along with the above, the maximum likelihood estimate of a normal distribution of each subsequence is also shown. This characteristic has also been observed by other authors [44,45,79].

All histograms shown in Figure 13 appear more pointed at the origin and heavier-tailed than the fitted Gaussian distributions. This shows that the subsequences contained a spatial structure with relatively smooth areas separated by abrupt transitions. Therefore, the wavelet coefficients had values near to zero in these uniform regions and were of great relative value in the transition zones, which were small when speaking in comparative terms of the areas. The above could be a reason that would justify the shape of these distributions.

The density functions of the marginal wavelets can be modeled using extreme value parametric distributions [80], whose parameters are related to the mean, variance, skewness, and kurtosis. Therefore, these moments are the characteristics of the distributions of the coefficients at the different levels of decomposition.

In this research, 56 values were obtained taking into account the following: the eight estimators described in the Materials and Methods Section (i.e., the mean, standard deviation, skewness, kurtosis, minimum, maximum, and 2nd and 98th centiles) for the full year, the marginal distributions of the detail coefficients of the N=5 levels, and the approximation coefficients of the fifth level. Additionally, for joint distributions, the entire subsequence of three regressions were considered. Therefore, the total number of these statistics was equal to 24.

Due to what is stated in this subsection, there were four subsequences of size $2^{11}=2048$ each for the series of each year. These subsequences represent the quarters of a year. From here, a feature vector with 56+24=80 components was obtained. Therefore, each year was characterized by a vector of dimension 80·4=320.

#### 3.4.1. Comparison between Feature Vectors by Using Principal Component Analysis

**Figure 14:**
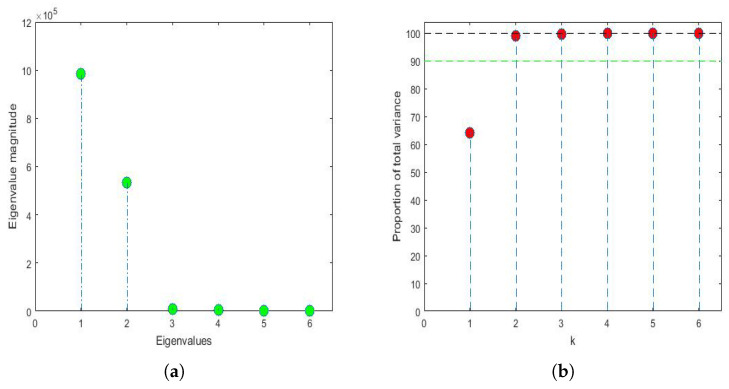
First eigenvalues and cumulative variance explained with the first eigenvalues. (**a**) First eigenvalues. (**b**) Proportion of total variance.

Applying the same philosophy of analysis explained by the authors in previous research [43,81], when analyzing the years under study, it was found that, for n=6 and p=320, the first three eigenvalues were 983,811, 533,076 and 8910 and that, from the fifth eigenvalue onward, the eigenvalues were equal to zero because only 6 years were analyzed (i.e., n=6). The first eigenvalues and the accumulated variability are shown in Figure 14. Thus, by keeping only the first two principal components, 98.95% of the variability was collected. In the same way, by keeping only the first three principal components, then 99.53% of the variability was collected.

**Figure 15:**
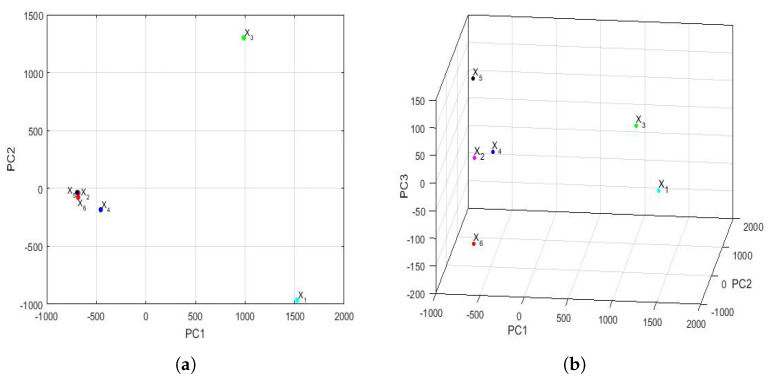
Location of the feature vectors of the variables with respect to the first two and three principal components: $X_{1}$ (2015), *…*, $X_{6}$ (2020). (**a**) Feature vectors: first 2 principal components. (**b**) Feature vectors: first 3 principal components.

The feature vectors [43] of the 6 years that were studied are shown in Figure 15, being the axes of such a figure the first principal components. In the two-dimensional graph (Figure 15a), it is observed, on the one hand, that the points appear to form the vertices of an isosceles triangle, where one of these vertices is made up of a grouping of the feature vectors of the variables $X_{2}$, $X_{4}$, $X_{5}$, and $X_{6}$. On the other hand, it is observed that the other two variables (i.e., $X_{1}$ and $X_{3}$) are the other two vertices of the isosceles triangle. Furthermore, when analyzing the three-dimensional graph (Figure 15b), it is noticed that the feature vectors of the variables $X_{1}$ and $X_{3}$ appear equidistant from all the others and that the feature vectors of the variables $X_{2}$ and $X_{4}$ are very close and located in a plane, together with the feature vectors of the variables $X_{1}$ and $X_{3}$. Moreover, the feature vectors of the variables $X_{5}$ and $X_{6}$ lie on both sides of the aforementioned plane.

Taking into account all that has been said above, it can be concluded that the variable $X_{6}$ (i.e., year 2020) had different characteristics from the rest of the years, as did the variable $X_{5}$. This happened because the plane determined by the feature vectors of the first four years (i.e., the feature vectors of the variables $X_{1}$, $X_{2}$, $X_{3}$, and $X_{4}$) separated $X_{5}$ and $X_{6}$ from the other variables. Finally, an analogy appeared between the years corresponding to the variables $X_{2}$ and $X_{4}$.

#### 3.4.2. Comparison between Feature Vectors by Using Multidimensional Scaling

**Figure 16:**
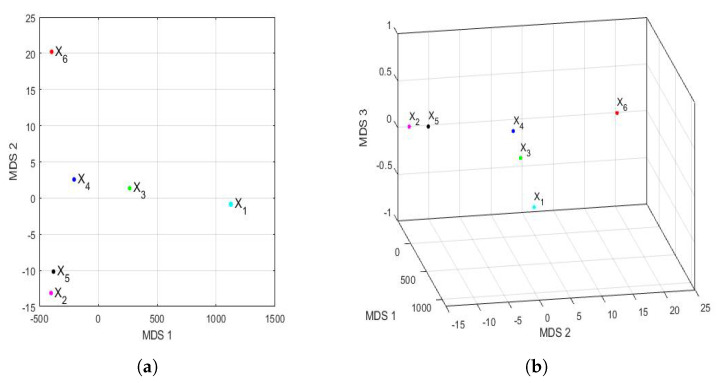
Location of the feature vectors of the variables with respect to the first two and three principal coordinates: $X_{1}$ (2015), *…*, $X_{6}$ (2020). (**a**) Feature vectors: first 2 principal coordinates. (**b**) Feature vectors: first 3 principal coordinates.

The Hausdorff distance and the Euclidean distance between the vectors that characterize the PM${}_{2.5}$ concentration of each year under study are shown in Table 9. The distances between the feature vectors, in general, preserved the order, but the magnitudes of these differences were very remarkable. Figure 16 shows the principal coordinate graphs of the vectors that characterize the PM${}_{2.5}$ concentration by years, when performing the nonmetric MDS with the Hausdorff distance between the feature vectors. Furthermore, the order of the stress Equation (3) was $S=2.9\cdot 10^{-16}$, which turned out to be excellent following the criteria established in other research [72].

Based on the results, when comparing Figure 15 and Figure 16, it can be said that the graphs based on the nonmetric MDS allow a better differentiation of the set of all years under study than the graphs based on the PCA. In the case of the nonmetric MDS, the conclusions obtained with the two-dimensional graph (Figure 16a) were the same as those obtained with the three-dimensional graph (Figure 16b), since in the latter, all the points seem to be located on the same plane. This plane seems to be determined by an origin point, which is the feature vector of the variable $X_{4}$, an axis consisting of the feature vectors of the variables $X_{1}$ and $X_{3}$, and another axis consisting of the feature vectors of the variables $X_{2}$, $X_{5}$, and $X_{6}$.

According to this procedure, the most similar years were 2016 ($X_{2}$) and 2019 ($X_{5}$). Additionally, the year 2020 ($X_{6}$), as with the PCA method, was far from all the others, because the horizontal axis separates the feature vector associated with $X_{6}$ from all the others. Moreover, the years 2016 ($X_{2}$) and 2019 ($X_{5}$) were similar to each other because they formed the only appreciable grouping. Having said this, the only difference with respect to the conclusions obtained with the PCA was that the variable $X_{2}$ was more similar to the variable $X_{5}$, instead of $X_{4}$ (see Figure 15). The rest of the conclusions, such as that $X_{1}$, $X_{3}$, and $X_{6}$ were different from all the other variables, continued to hold.

#### 3.4.3. Additional Comparative Analysis by Semesters

At this point, it is worth remembering that the data that were completed for each semester were for the same month, day, and time for each of the six years. Furthermore, given that 4096 observations were available for each semester, it was decided to make the same comparisons previously made for each semester. That is, a comparison was made between the first semester of the 2015–2020 six-year term and the second semester of the same six-year term. Likewise, it is worth remembering that, in the first three months of 2020, there were no changes in the historic center of the city of Quito that could be attributed to the restrictions due to the coronavirus disease (COVID-19).

As proven in this section (Section 3.4), for each semester, there was a vector that characterized it of dimension 320, and by subdividing the series that forms each semester into four parts, it returned to have 80 values that characterized each of the subdivisions.

That said, when performing the principal component analysis of the six years under study, for the first semester of each year, it was found that more than 99% of the variability of the observations could be explained with the first two principal components. Likewise, 87.8% of the variability of the observations of the second semester of the six years under study could be explained with the first three principal components.

**Figure 17:**
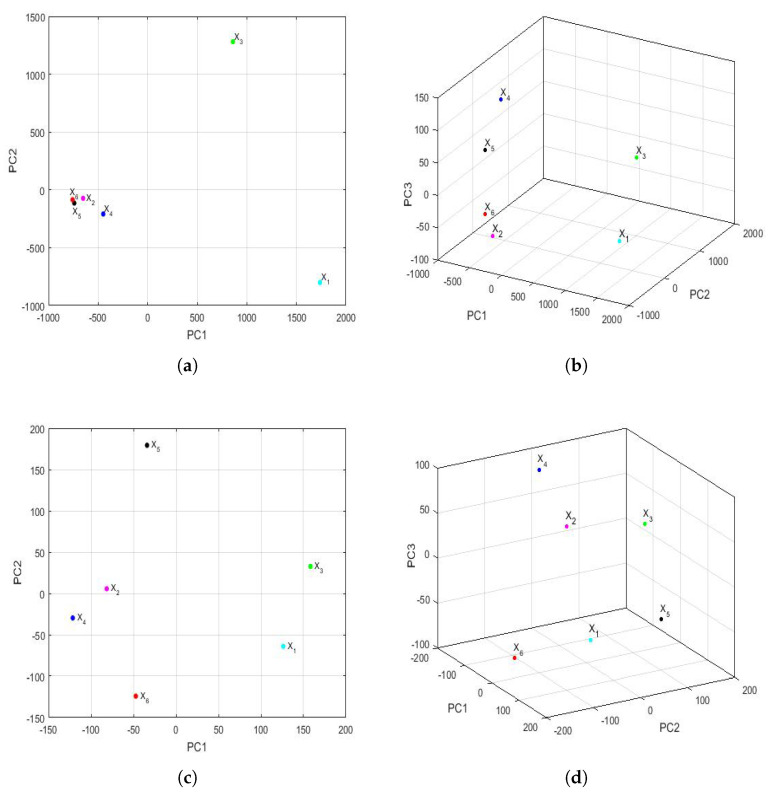
First two and three principal components of the feature vectors by semesters of each of the years of the 2015–2020 six-year term: $X_{1}$ (2015), *…*, $X_{6}$ (2020). (**a**) Feature vectors: first 2 principal components of the first semester. (**b**) Feature vectors: first 3 principal components of the first semester. (**c**) Feature vectors: first 2 principal components of the second semester. (**d**) Feature vectors: first 3 principal components of the second semester.

Figure 17 shows the representation of the feature vectors for each of the years of the 2015–2020 six-year term, with respect to the first two principal components and the first three principal components. In addition, the above was performed for both the first semester and the second semester of each year.

Considering the scale, Figure 17 shows many more differences in the first semester than in the second semester. Additionally, considering the graph in two dimensions (shown in Figure 17a), for the first semesters, a great resemblance to the same graph can be seen for the years (see Figure 15a). Here, the points are located at the vertices of a triangle, and in one of the vertices, there is a grouping that consists of the years 2016 ($X_{2}$), 2018 ($X_{4}$), 2019 ($X_{5}$), and 2020 ($X_{6}$).

On the other hand, when observing the three-dimensional graph of the first semester (shown in Figure 17b), it can be seen that there are differences from Figure 15b. In this case, the differences between the four years grouped in the two-dimensional graph (shown in Figure 17a) are smaller, and no great differences are seen in the year 2020 ($X_{6}$) compared with the others.

The above changed remarkably when analyzing the results of the second semester. The scale of the observations shown in Figure 17c,d allowed us to better appreciate the discrepancies between the second semesters of all years. In fact, all the points appear to be points on a sphere whose center is equidistant from all of them. Now, the second semester of 2020 ($X_{6}$) differed from the second semesters of the rest of the years, and there were no groupings between the feature vectors that were considered.

**Figure 18:**
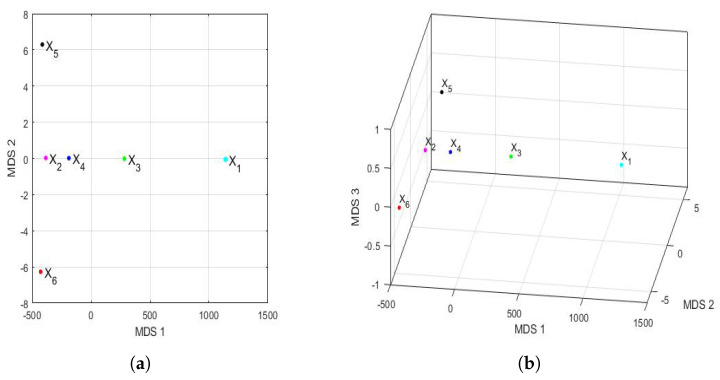

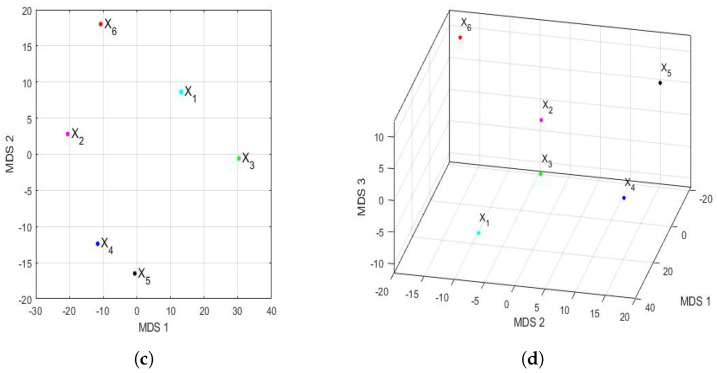
First two and three principal coordinates of the feature vectors by semesters of each of the years of the 2015–2020 six-year term: $X_{1}$ (2015), *…*, $X_{6}$ (2020). (**a**) Feature vectors: first 2 principal coordinates of the first semester. (**b**) Feature vectors: first 3 principal coordinates of the first semester. (**c**) Feature vectors: first 2 principal coordinates of the second semester. (**d**) Feature vectors: first 3 principal coordinates of the second semester.

At this point, we carried out the analysis of the disparities in the PM${}_{2.5}$ concentration in the 2015–2020 six-year term by semester by using MDS. In order to do this, Figure 18 shows the representation of the feature vectors for each of the years of the 2015–2020 six-year term, with respect to the first two principal coordinates and the first three principal coordinates. In addition, as in Figure 17, the above was performed for both the first semester and the second semester of each year. In this case, the appraisals for the first semester (see Figure 18a,b) were relatively similar to those made for the full years (see Figure 16). Furthermore, the order of the stress Equation (3) was $2\cdot 10^{-16}$ for both the two-dimensional and the three-dimensional cases, which turned out to be excellent [72].

Additionally, as in Figure 16, the two-dimensional graph does not provide more information than the three-dimensional graph. Figure 18a,b show that the vectors are located in a plane determined by two practically orthogonal axes. Moreover, the differences from Figure 16 are as follows: (1) the points are more concentrated than in the case of the years, and (2) the point $X_{2}$ (2016) appears displaced in the second coordinate. Moreover, as with PCA, the differences in the conclusions by years were obtained for the second semester (see Figure 18c,d). The first difference was that the two-dimensional fit was acceptable ($S=11\cdot 10^{-2}$), and the three-dimensional fit was excellent ($S=6\cdot 10^{-7}$). In addition, the concentration of values in the first coordinate is much higher for the second semester graph than in the year graph (see Figure 16).

Finally, the second half of 2020 ($X_{6}$) was clearly distinguishable from the rest of the years (see Figure 18c,d), and now, there seemed to be a certain similarity between the second half of 2016 ($X_{2}$) and 2017 ($X_{3}$) (see Figure 18d). Moreover, the graph of the points for the second semester also seems to suggest that all the points are equidistant from a point that would be the center of a sphere.

#### 3.4.4. Additional Comparative Analysis by Trimesters

In this case, the four parts into which the 8192 observations for each year were separated did not strictly correspond to the trimesters, although half of the observations did correspond to the semesters. In addition, the same analysis that was carried out for years and for semesters is carried out in this subsubsection, remembering that, in the first months of 2020, the PM${}_{2.5}$ concentration at the air quality monitoring station under study was not affected by causes due to COVID-19. Furthermore, now, each feature vector had 320 elements, but these 320 values characterized a series of 2048 observations, while in the case of years, they characterized a series of 8192 observations, while in the case of semesters, they characterized a series of 4096 observations.

**Figure 19:**
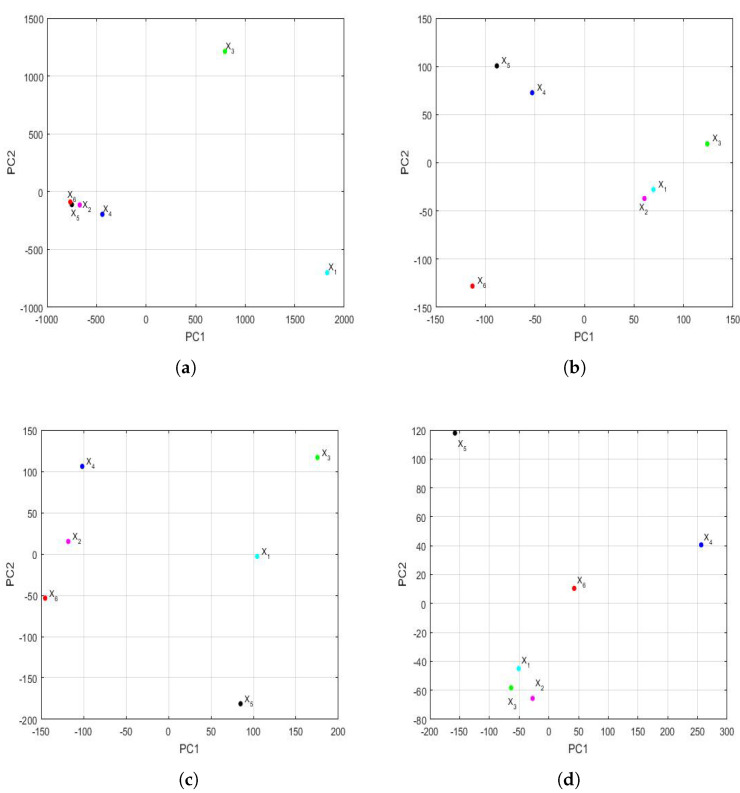
First two principal components of the feature vectors by trimesters of each of the years of the 2015–2020 six-year term: $X_{1}$ (2015), *…*, $X_{6}$ (2020). (**a**) Feature vectors: first 2 principal components of the first trimester. (**b**) Feature vectors: first 2 principal components of the second trimester. (**c**) Feature vectors: first 2 principal components of the third trimester. (**d**) Feature vectors: first 2 principal components of the fourth trimester.

Figure 19 shows the graph of the first principal components of the feature vectors for the trimesters of each of the years. This figure shows the great difference in magnitude that existed in terms of the differences between the variables in the first trimester in relation to all other trimesters. Now, the variability explained by the first two principal components was as follows: 99.5% in the first trimester, 77.9% in the second trimester, 70.5% in the third trimester, and 83.2% in the fourth trimester.

In addition, Figure 19 shows that, in the second trimester, it can already be seen that the year 2020 ($X_{6}$) differed from the rest of the years in each trimester. Apart from the first trimester, the groupings appeared to occur between the variables $X_{1}$ (2015) and $X_{2}$ (2016) in the second trimester and the variables $X_{1}$ (2015), $X_{2}$ (2016), and $X_{3}$ (2017) in the fourth trimester. Here, it should be noted that the third trimester graph appears equidistant from a point in the center of all of them.

**Figure 20:**
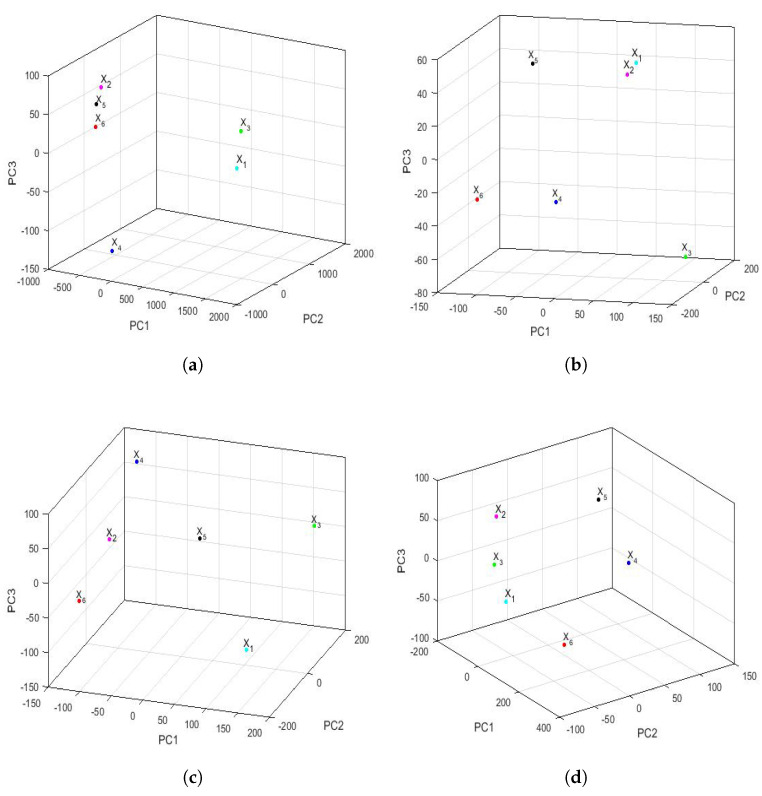
First three principal components of the feature vectors by trimesters of each of the years of the 2015–2020 six-year term: $X_{1}$ (2015), *…*, $X_{6}$ (2020). (**a**) Feature vectors: first 3 principal components of the first trimester. (**b**) Feature vectors: first 3 principal components of the second trimester. (**c**) Feature vectors: first 3 principal components of the third trimester. (**d**) Feature vectors: first 3 principal components of the fourth trimester.

Figure 20 shows the analysis carried out by PCA of the feature vectors of the trimesters in three dimensions. In some cases, three-dimensional graphical representations were more accurate than two-dimensional graphical representations. These graphs, which explain 99.8%, 90.4%, 87.4%, and 95.1% of the variability of the feature vectors for each semester, respectively, corroborate the greater distance that existed between the feature vectors in the first trimester than in the other trimesters. In addition, Figure 20 also shows that the first trimester of 2020 ($X_{6}$) was similar to the first trimester of 2016 ($X_{2}$) and 2019 ($X_{5}$) (see Figure 20a). Furthermore, in the third and fourth trimester graphs (shown in Figure 20c,d), the variable corresponding to the year 2020 is clearly seen to be far from the others. Moreover, in the third trimester, the arrangement of the points is similar to the arrangement of the points in Figure 17d, which corresponds to the analysis of the differences by semester. In short, the points appear to be located on a sphere with a center equidistant from all of them. Besides, drawing one more conclusion about the fourth trimester, it can be said that there existed only similarities between the points of the variables $X_{1}$ (2015), $X_{2}$ (2016), and $X_{3}$ (2017).

To finalize the study of the differences between the feature vectors of the trimesters, the graphs obtained through the analysis carried out using the nonmetric MDS were considered. In this case, the stress values were excellent, being $S<1.6\cdot 10^{-5}$ in both two and three dimensions.

**Figure 21:**
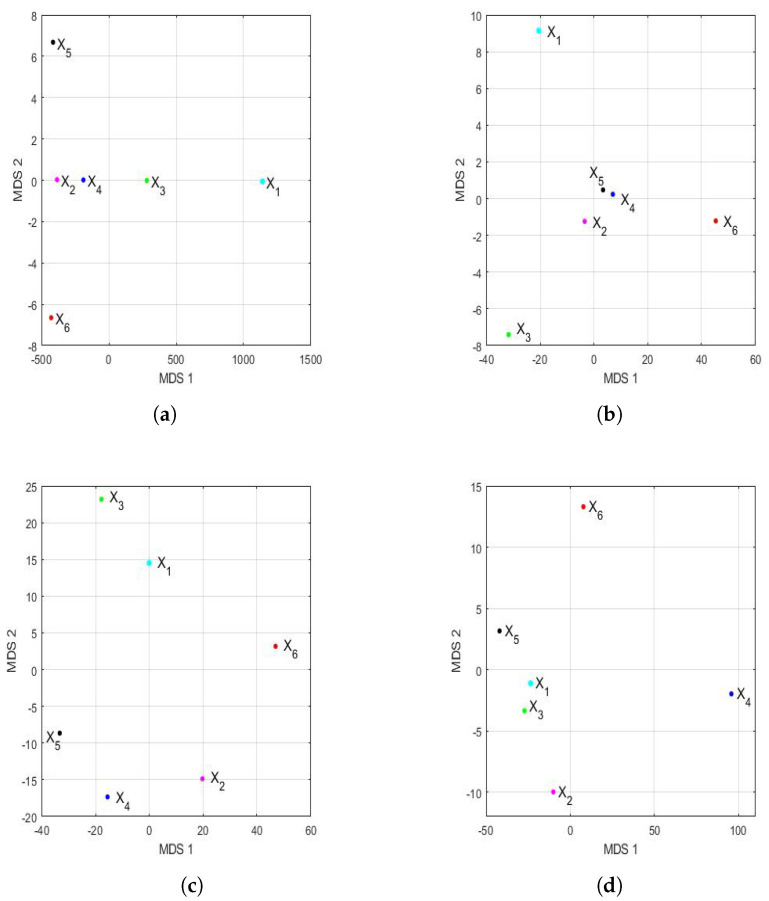
First two principal coordinates of the feature vectors by trimesters of each of the years of the 2015–2020 six-year term: $X_{1}$ (2015), *…*, $X_{6}$ (2020). (**a**) Feature vectors: first 2 principal coordinates of the first trimester. (**b**) Feature vectors: first 2 principal coordinates of the second trimester. (**c**) Feature vectors: first 2 principal coordinates of the third trimester. (**d**) Feature vectors: first 2 principal coordinates of the fourth trimester.

**Figure 22:**
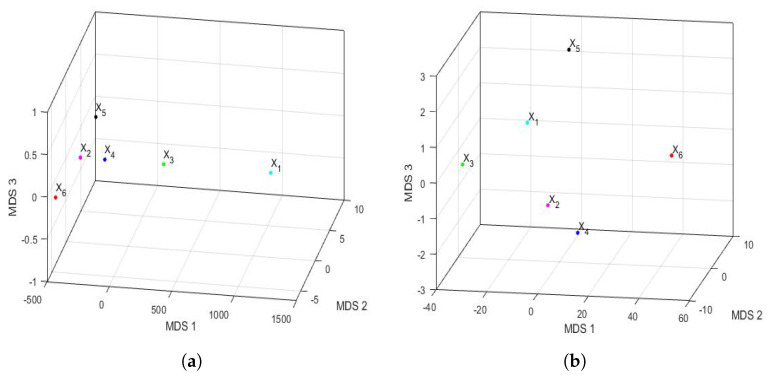

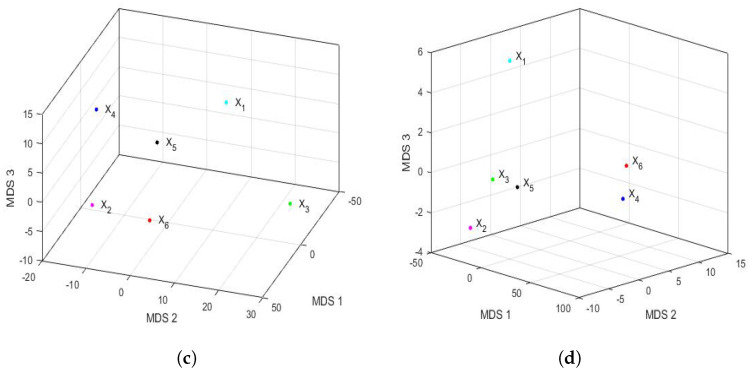
First three principal coordinates of the feature vectors by trimesters of each of the years of the 2015–2020 six-year term: $X_{1}$ (2015), *…*, $X_{6}$ (2020). (**a**) Feature vectors: first 3 principal coordinates of the first trimester. (**b**) Feature vectors: first 3 principal coordinates of the second trimester. (**c**) Feature vectors: first 3 principal coordinates of the third trimester. (**d**) Feature vectors: first 3 principal coordinates of the fourth trimester.

Figure 21 shows the analysis carried out by the nonmetric MDS of the feature vectors of the trimesters in two dimensions. It is observed that Figure 21a is similar to Figure 18a. In addition, as with PCA, there was a large difference in the differences between the first trimester measurements versus the other trimesters. What is more, it was observed that 2020 ($X_{6}$) was very far from the other years. Furthermore, joining the analysis with the graphs in Figure 22, it can be concluded that the variables $X_{2}$ (2016) and $X_{4}$ (2018) were both similar in the second trimester and far from all the other variables in that trimester (see Figure 22b). Moreover, in the third trimester, the variables seem to be located in a sphere with a center equidistant from all the points (see Figure 22c). Besides, in the graphs corresponding to the fourth trimester (see Figure 21d and Figure 22d), the variables $X_{4}$ (2018) and $X_{6}$ (2020) are clearly far from the others, and the variables $X_{2}$ (2016), $X_{3}$ (2017), and $X_{5}$ (2019) can be grouped together.

### 3.5. On Main Air Pollutants in Quito and Influence of the “Burning of the Old Year” Festive Activity in the PM${}_{2.5}$ Concentration

According to what is presented in the Quito air quality reports that were issued by the Quito Environmental Protection Agency from 2003 to 2019 (http://www.quitoambiente.gob.ec/index.php/informes (accessed on 30 September 2022) ), it is transport that fundamentally affects air pollution in Quito, especially the transport of light and heavy vehicles that use diesel fuel. In addition, there is substantial vehicular congestion, several thermoelectric plants, and several industries that use bunker and fuel oil for their operation. However, the measures taken by the Government of Ecuador during the lockdowns (https://www.gestionderiesgos.gob.ec/wp-content/uploads/2020/03/Informe-de-Situación-No008-Casos-Coronavirus-Ecuador-16032020-20h00.pdf (accessed on 30 September 2022) ), to prevent community transmission of COVID-19, also considerably reduced transportation and the operation of factories and industries, in general.

At this point, it is worth making a few additional important comments about the characteristics of the data. Specifically, Figure 3a shows that the number of anomalous observations in January was large compared with the rest of the months of the year. Regarding this, the authors of this paper think that this was due to the cultural heritage and historical tradition of Ecuador. Specifically, every year on 31 December, one or two minutes before the chimes of the beginning of the New Year, with joy and in union with family and friends, the Burning of the Old Year is carried out. During this festive activity, a figure representing the year that ended is burned. This burning lasts at least one hour, and this is done as a symbol of hope and closure of a cycle that ends. In short, the essence is to burn everything bad that could have happened in the year that has ended and to maintain the illusion and hope of better days in the year that has just begun (https://www.turismo.gob.ec/la-quema-del-ano-viejo-en-ecuador-una-tradicion-llena-de-emotividad-y-picardia (accessed on 30 September 2022) ).

### 3.6. Summary

In summary, within the framework of pattern recognition, a computational tool applied to the analysis of the differences, or classification, of the concentration of PM${}_{2.5}$ in the region under study was presented. This tool made it possible to visually reflect a measure of the distance between years during the 2015–2020 six-year term, based on all the data collected during that time period.

Here, after carrying out a previous statistical analysis of the data, it was found that observations were missing in a non-random context, because these observations were missing consecutively during hours of the same day, even during all hours of the day. In addition, the above must be added to the fact that there were very few missing observations in isolation.

Furthermore, the number of observations with high PM${}_{2.5}$ concentration was approximately 3% of the total observations, indicating that the observations may have come from heavy-tailed distributions. Moreover, this large number of observations could have been due to the existence of a mixture of distributions, although in the analyses that the authors carried out when studying other series of polluting materials, this hypothesis is less likely [1,59,60].

Due to the complexity in the distribution of the observations and the structure of the missing values, in this research, it was decided to fill in some missing values in a few hours of each semester to achieve a quantity that would be a power of two. This was done for technical reasons, in order to apply the transforms efficiently. Therefore, the data analysis was carried out guaranteeing the existence of a sufficient number of observations to perform the study of the data by hours of each month and year efficiently.

To the above, it must be added that this study took into account the fact that, as a consequence of the COVID-19 pandemic, in 2020, there were restrictions that reduced some of the causes that provoke the emission of particulate matter in the area where the measurements were made. Therefore, it was decided not to fill in any of the missing data in 2020.

In order to be able to fill in the missing data with plausible values, different datasets related to each of the missing data were built and different interpolation methods were applied, together with classical and robust statistics. Then, to test the quality of the proposed method to fill in the missing data, a month was taken as the test month, and several comparisons were made with different observations that were purposely made to disappear by the authors of this paper in the chosen test month. This month was March 2019, and it was taken as a test month because, in that month, there was only one missing observation. Therefore, this month was used to fill in some observations following the behavior patterns of the data and, thus, be able to assess the characteristics and qualities of the proposed method.

Once this was done, 8192 observations were available for each year of study and 4096 each semester. Thus, following general pattern recognition methods, it was decided to extract a feature vector for each time period that was considered (i.e., year, semester, and trimester). The dimension of this vector was equal to 320. Naturally, there were fewer nuances by years than by quarters, because despite having the same dimensions, the time series in each case had different lengths. Next, to analyze the differences between the feature vectors that were built, two techniques were used: PCA and nonmetric MDS. In addition, during the application of the MDS, once the feature vectors were built, the Hausdorff distance between said vectors was applied. In short, the choice of the Hausdorff distance was due to the fact that many distances depend on the variability of the measurements, while the Hausdorff distance is a metric by which the degree of similarity between two vectors is determined.

To make the graphical representations, figures were used that represented in the same two-dimensional or three-dimensional graph both the objects under study (i.e., years, semesters, and trimesters) and the variables (i.e., feature vectors). That said, since PCA retains the greatest variability through the first principal components, through linear combinations of the orthogonal variables, the graphical representations showed the differences between the analyzed units (i.e., years, semesters, and trimesters) using the point of view of the dispersion of the variables.

On the other hand, by using MDS, the data were transformed into points that represented the distance between them, and the differences between real distances and adjusted distances were minimized. It is worth mentioning that, in the case of the Euclidean distances, there is a duplication between MDS and PCA. However, in the case of considering similarities instead of distances, the nonmetric MDS is preferable because it tries to reproduce those similarities. Unlike PCA, with nonmetric MDS, only the lack of similarity is taken into account. Therefore, the variability between the observations was not considered.

The results of the analysis carried out for the annual series of the six-year period that was studied (i.e., 2015–2020) confirmed the difference that existed between the PM${}_{2.5}$ concentration in 2020 and the PM${}_{2.5}$ concentration in the 2015–2019 five-year period. These differences became more evident through the analysis that was performed by using the nonmetric MDS, because sometimes, it was enough to use the two-dimensional representation to be able to appreciate the significant differences that existed between the periods corresponding to some years with respect to others. However, on the other hand, in order to appreciate these differences with PCA, it was necessary to carry out the three-dimensional representation on several occasions to be able to reaffirm that there were some discrepancies between the different periods of time that were studied.

The above-mentioned differences had different scales, which were greater when the PCA was used than when the nonmetric MDS was used. In general, here, it was possible to separate more and better the units that were analyzed by using nonmetric MDS-based graphs than by using PCA-based graphs. Regarding this result, the authors of this paper think that this is so because, with PCA, the variability of the data is retained in an orthogonal way, and both the study elements and their characteristics are represented. On the other hand, the nonmetric MDS only tries to reproduce the distance that exists between the study elements, without trying to retain the variability of the data in an orthogonal way. The differences between the results yielded by using both techniques (i.e., PCA and nonmetric MDS) have already been mentioned by other authors [82].

Regarding the results of the analysis of the semesters and trimesters, the PCA showed that the difference in PM${}_{2.5}$ concentration between the first semester of 2020 and the first semester of the other years was less appreciable than the difference in PM${}_{2.5}$ concentration between the second semester of 2020 and the other years. Additionally, the nonmetric MDS showed that there was a clear difference between the PM${}_{2.5}$ concentration in 2020 and the other years, both in the first semester and in the second semester. Furthermore, it is clear that this technique only needed to work in two dimensions to show the clear differences that existed between the analyzed semesters. Moreover, the results of the analysis by trimesters, using the described procedures, reaffirmed the difference between 2020 and the other years. This difference became more noticeable from the second trimester onward. Besides, in the first trimester, the magnitudes of the differences were much greater when using the nonmetric MDS than when using the PCA.

On the other hand, there were clear differences between the rest of the trimesters by using both procedures. However, once again, with the nonmetric MDS, it was possible to better appreciate the difference that existed between trimesters. In this sense, even working in two dimensions was enough to appreciate the clear differences between the analyzed trimesters by using the nonmetric MDS.

In light of the forgoing, it can be said that the measures taken by governments to curb the spread of the coronavirus disease (COVID-19) [17,18] also helped cities reduce air pollution due to particulate matter [19,20,21,22,23,24,25,26,27,28,29].

Finally, and unfortunately, variants of the virus (SARS-CoV-2) continue to affect many countries. However, the international scientific community is working tirelessly in search of vaccines that offer effective protection against variants of the virus and that avoid the need for new lockdowns [83,84].

## 4. Conclusions

In this research, a robust method to carry out the PM${}_{2.5}$ concentration analysis in a study region was presented. The method imputed missing data in time series and combined a statistical analysis, which included robust statistics, with the wavelet decomposition of the data to be compared. The proposed method treated the data as objects, and it found important differences between possibly similar objects. All this was done by using a proposed technique for object characterization, and both principal component analysis and multidimensional scaling were used to compare the variables of interest.

In essence, two different ways were used to test that the estimated data from the missing observations were the best. One way was to use different interpolation methods, and the other way was to use classical and robust statistics. Here, it was discovered that, when the missing data sequences were of a small size (missing data sequences, runs, of lengths less than 10), it was better to use interpolation methods. However, when the size of the runs of missing observations was greater than 10, then it was better to use classical and robust statistics.

It is important to mention that eight different non-disjoint sets were proposed to complete the data. In addition, the eight sets were not all two-by-two disjoint, because we sought to maximize redundancy in the data analysis. This redundancy was good, because it brought out intrinsic features of the data. Then, these eight sets were the ones that were subjected to the above-mentioned two different ways of testing, to find the best data filling method. Therefore, in the end, the best form, method, and statistics were proposed according to the case.

In this research, the classification of PM${}_{2.5}$ concentration was based on object identification using feature vectors, and then, the separation between objects was measured using a metric. To obtain the feature vectors, the data sequences were decomposed using a multiscale decomposition of the Daubechies discrete wavelet family. In addition, the statistics of marginal and joint distributions were used to categorize the data. Finally, the differences between the feature vectors were studied using the PCA and MDS techniques. The first technique was applied using distance-based correlation coefficients between the feature vectors. On the other hand, the second technique was applied using a distance based on the Hausdorff distance.

Here, the hypothesis to be tested was that the lockdown in downtown Quito during 2020 produced a significant drop in PM${}_{2.5}$ concentration throughout that year compared with the years 2015–2019. The results of the analysis supported the hypothesis and showed that there were significant differences.

Having the scientific analysis of data related to air pollution is of the utmost importance for citizens and decision-makers at the local, national, and international levels. This can establish positive changes in society. For example, for the case at hand, we think that the lockdowns during the first year of the pandemic might have reduced the number of deaths from air pollution in cities. Additionally, we believe that the reduction in air pollution, due to the lockdowns, was a positive factor in the decrease in preventable non-communicable diseases.

Some examples of things that can be done to reduce air pollution in cities are as follows. (1) Urban decision-makers can promote research projects aimed at the construction of smart parks intended to function as urban pollution filters, (2) In addition, the means used to keep citizens informed in real-time about pollution levels in different areas of cities can be improved by using Internet of Thing devices and technologies. (3) Furthermore, intelligent systems and green spaces can be built to protect citizens from pollution. (4) Moreover, it is a well-known fact that air quality can be improved by building and using more efficient and environmentally friendly transport based on renewable and sustainable energies. Everything said above highlights the importance of putting scientific research at the service of society, to guarantee a better quality of life for all citizens.

As future research, we plan to obtain a robust model of the dynamic behavior of the PM${}_{2.5}$ concentration in downtown Quito. The model will take into account at least the last five years.

## Figures and Tables

**Table 1:** Data distribution.

Year	Semester	Number of Data	Number of Null Observations	Number of Negative Observations	Number of Missing Data	Number of Valid Observations
2015	First	4344	17	0	27	4300
	Second	4416	5	0	30	4381
2016	First	4344	4	0	151	4189
	Second	4416	1	18	510	3887
2017	First	4344	1	20	227	4096
	Second	4416	7	0	205	4204
2018	First	4344	9	0	252	4083
	Second	4416	11	0	201	4204
2019	First	4344	18	0	333	3993
	Second	4416	3	0	162	4251
Total	First	21,720	49	20	990	20,661
	Second	22,080	27	18	1108	20,927
2020	First	4344	20	0	45	4279
	Second	4416	34	0	81	4301

**Table 2:** Summary statistics.

Year	Count	Mean	Median	Standard Deviation	Skewness	Kurtosis	Minimum	Maximum	Outliers %
2015 ($X_{1}$)	8681	15.8902	13.7500	13.9598	13.1837	399.4601	0.02	524.04	2.94
2016 ($X_{2}$)	8076	17.3427	15.5200	10.6226	1.2743	6.0232	0.01	103.44	2.66
2017 ($X_{3}$)	8300	17.9939	15.8900	18.2636	24.3487	1008.0373	0.06	837.34	3.11
2018 ($X_{4}$)	8287	17.1833	15.5600	10.7868	3.5491	59.7379	0.03	276.67	2.68
2019 ($X_{5}$)	8244	15.5926	13.7600	10.6252	4.2567	65.1195	0.01	248.10	3.04
2020 ($X_{6}$)	8580	12.8333	11.2200	8.5752	1.5644	9.6861	0.01	127.45	2.66
Total	50,168	16.1139	14.2000	12.6615	16.2533	842.3658	0.01	837.34	2.85

**Table 3:** Test results, values of the constant *K*, and *p*-value for each meteorological variable.

	January	February	March	April	May	June	July	August	September	October	November	December
WS	0	0	0	0	0	0	0	0	0	0	0	0
*K*	0.1	0.1	0.1	−0.1	0	−0.2	−0.3	−0.6	−0.3	0.3	0	−0.1
*p*-value	0.642	0.464	0.694	0.780	0.795	0.903	0.598	0.812	0.086	0.951	0.951	0.583
HR	0	0	0	0	0	0	0	0	0	0	0	0
*K*	0	−2	1	0	−1	5	8	10	2	−8	−10	3
*p*-value	0.489	0.104	0.191	0.093	0.156	0.267	0.201	0.181	0.258	0.086	0.064	0.532
R (R > 0)	0	0	0	0	0	0	0	1	0	0	0	0
*K*	0	0	0	0	0	0	0	0	0	0.2	0	0
*p*-value	0.175	0.390	0.806	0.368	0.465	0.516	0.169	0	0.349	0.114	0.818	0.073
SI	0	0	0	0	0	1	0	0	0	0	0	0
*K*	0.01	0.01	0.01	0.01	0.01	0.001	0	0	0	0	0	0
*p*-value	0.458	0.831	0.066	0.627	0.379	0.045	0.646	0.716	0.230	0.438	0.920	0.060
WD	0	0	0	0	0	0	0	0	0	0	0	0
*K*	0	3	−5	0	0	0	−1	−18	3	2	25	0
*p*-value	0.672	0.067	0.083	0.809	0.749	0.466	0.075	0.069	0.078	0.156	0.080	0.086
T	0	0	0	0	0	0	0	0	0	0	0	0
*K*	0	0	0	0	0.4	−0.4	−0.5	−0.2	−0.4	0.5	0	−0.6
*p*-value	0.239	0.167	0.791	0.243	0.159	0.056	0.153	0.078	0.108	0.160	0.416	0.116
AP	0	0	0	0	0	0	0	0	0	0	0	0
*K*	0.3	0.3	0.5	0.7	0.5	0.2	0	−0.1	0	0	0.5	0.4
*p*-value	0.406	0.127	0.525	0.112	0.230	0.052	0.282	0.340	0.353	0.320	0.425	0.085
UV	0	0	0	0	0	0	0	0	0	0	0	1
*K*	0	0	0	0	0	0	0	0	0	0	0	0
*p*-value	0.423	0.256	0.075	0.200	0.072	0.264	0.883	0.762	0.996	0.213	0.813	0.020

**Table 4:** December 2019 vs. December 2020: missing data and non-missing data. P1: From 0:00 on 1 December to 11:00 on 19 December. P2: From 12:00 on 19 December to 23:00 on 31 December.

	P1: Non-Missing Data	P1: Missing Data	P1: Total	P2: Non-Missing Data	P2: Missing Data	P2: Total
2019	415 (93.47%)	29 (6.53%)	444	298 (99.93%)	2 (0.67%)	300
2020	18 (4.05%)	426 (95.95%)	444	298 (99.93%)	2 (0.67%)	300

**Table 5:** Evaluation of models with 111 missing data.

Estimation	Pearson’s *r*	Kendall’s τ	Spearman’s ρ	RSS
$Mean^{D}$	0.414	0.278	0.416	8618.99
$T_{wa}^{D}\left(c\right)$	0.402	0.270	0.401	8800.63
$Mean^{W}$	0.396	0.241	0.362	8918.15
$Me^{W}$	0.428	0.283	0.414	9454.69
$T^{W}\left(0.1\right)$	0.420	0.248	0.373	8923.16
$T^{W}\left(0.2\right)$	0.403	0.249	0.367	9432.26

**Table 6:** Evaluation of models with 64 missing data.

Estimation	Pearson’s *r*	Kendall’s τ	Spearman’s ρ	RSS
GE	0.528	0.419	0.587	8451.75
$T_{wa}^{M}\left(2.4\pi \right)$	0.515	0.410	0.573	7901.15
$Me^{5}$	0.547	0.325	0.481	7390.41
$T_{wa}^{5}\left(2.4\pi \right)$	0.514	0.277	0.404	7046.48

**Table 7:** Evaluation of models with 49 missing data.

Interpolation	Pearson’s *r*	Kendall’s τ	Spearman’s ρ	RSS
Piecewise linear	0.519	0.372	0.534	3054.38
Nearest neighbor	0.520	0.345	0.474	3383.98
Piecewise cubic Hermite	0.502	0.351	0.508	3242.14
Modified Akima cubic Hermite	0.508	0.343	0.480	3893.92

**Table 8:** Evaluation of models with 33 missing data.

Estimation	Pearson’s *r*	Kendall’s τ	Spearman’s ρ	RSS
GE	0.263	0.156	0.201	1811.37
$Mean^{D}$	0.269	0.170	0.215	1443.25
$Me^{D}$	0.330	0.212	0.310	1571.71
$T^{D}\left(0.2\right)$	0.266	0.155	0.201	1331.56
$Mean^{W}$	0.257	0.186	0.256	1296.51

**Table 9:** Hausdorff and Euclidean distances between feature vectors of the years under study: 2015 ($X_{1}$), *…*, 2020 ($X_{6}$).

Distances		Hausdorff Distances					
		$X_{1}$	$X_{2}$	$X_{3}$	$X_{4}$	$X_{5}$	$X_{6}$
	$X_{1}$	0	1529.5	861.1	1333.3	1508.9	1524.7
	$X_{2}$	2394.5	0	668.4	196.1	20.6	33.7
Euclidean	$X_{3}$	2328.5	2141.7	0	472.3	647.8	663.6
distances	$X_{4}$	2133.6	305.9	2074.4	0	175.5	191.3
	$X_{5}$	2409.3	212.5	2153.8	367.4	0	34.0
	$X_{6}$	2386.1	213.6	2171.7	356.7	300.6	0

## Data Availability

Not applicable.
